# Evaluation of classification approaches for distinguishing brain states predictive of episodic memory performance from electroencephalography^✩^

**DOI:** 10.1016/j.neuroimage.2021.118851

**Published:** 2021-12-22

**Authors:** Soroush Mirjalili, Patrick Powell, Jonathan Strunk, Taylor James, Audrey Duarte

**Affiliations:** a Department of Psychology, University of Texas at Austin; b School of Psychology, Georgia Institute of Technology; c Department of Neurology, Emory University, Atlanta, GA, USA

**Keywords:** Classification, Episodic memory, Electroencephalography, Method comparison, Brain-computer interface

## Abstract

Previous studies have attempted to separate single trial neural responses for events a person is likely to remember from those they are likely to forget using machine learning classification methods. Successful single tria classification holds potential for translation into the clinical realm for real-time detection of memory and other cognitive states to provide real-time interventions (i.e., brain-computer interfaces). However, most of these studies—and classification analyses in general—do not make clear if the chosen methodology is optimally suited for the classification of memory-related brain states. To address this problem, we systematically compared different methods for *every* step of classification (i.e., feature extraction, feature selection, classifier selection) to investigate which methods work best for decoding episodic memory brain states—the first analysis of its kind. Using an adult lifespan sample EEG dataset collected during performance of an episodic context encoding and retrieva task, we found that no specific feature type (including Common Spatial Pattern (CSP)-based features, mean, variance, correlation, features based on AR model, entropy, phase, and phase synchronization) outperformed others consistently in distinguishing different memory classes. However, extracting all of these feature types consistently outperformed extracting only one type of feature. Additionally, the combination of filtering and sequential forward selection was the optimal method to select the effective features compared to filtering alone or performing no feature selection at all. Moreover, although all classifiers performed at a fairly similar level, LASSO was con sistently the highest performing classifier compared to other commonly used options (i.e., naïve Bayes, SVM, and logistic regression) while naïve Bayes was the fastest classifier. Lastly, for multiclass classification (i.e., levels of context memory confidence and context feature perception), generalizing the binary classification using the binary decision tree performed better than the voting or one versus rest method. These methods were shown to outperform alternative approaches for three orthogonal datasets (i.e., EEG working memory, EEG motor imagery, and MEG working memory), supporting their generalizability. Our results provide an optimized methodological process for classifying single-trial neural data and provide important insight and recommendations for a cognitive neuroscientist’s ability to make informed choices at all stages of the classification process for predicting memory and other cognitive states.

## 1. INTRODUCTION

For decades, researchers have been interested in distinguishing the neural activity associated with different cognitive states in various domains including motor imagery (reviewed in Ang & Guan, 2017; Fadiga & Craighero, 2004; Savaki & Raos, 2019), episodic and semantic memory (reviewed in Friedman & Johnson, 2000; Herweg, Solomon, & Kahana, 2020; Jaeger & Parente, 2008; Peigneux, 2015; T.-T. Wang, Mo, & Shu, 2009), emotion (reviewed in Dixon, Thiruchselvam, Todd, & Christoff, 2017; Lindquist, Wager, Kober, Bliss-Moreau, & Barrett, 2012; Phan, Wager, Taylor, & Liberzon, 2002), language (reviewed in Baciu & Perrone-Bertolotti, 2015; Beres, 2017; Brown & Hagoort, 2003; Friederici, 2004; Gabrieli, Poldrack, & Desmond, 1998), perception and attention (reviewed in Pessoa & Ungerleider, 2004; Taylor & Thut, 2012; Woodman, 2010), etc. The vast majority of these studies, using either functional magnetic resonance imaging (fMRI) or electroencephalography (EEG) methods, have used an averaging approach. That is, signals from many trials of a particular type are averaged together to increase signal to noise ratios and consequently, the power to detect the differences between two or more cognitive states (e.g., successful vs. unsuccessful memory, negative vs. neutral events). While the signal averaging approach provides important insight about neural differences between distinct cognitive states, a major limitation of this approach is that it does not allow for the exploration of single trials associated with cognitive states that might vary from moment to moment. For instance, consider episodic memory and the neural underpinnings of successful or unsuccessful encoding of different events. It is highly likely that not every event will be learned in the exact same way, with encoding strategy or depth varying between events; by using an averaging strategy, these neural differences between single events will be lost.

Exploration of brain states associated with single events—particularly when recorded from the scalp using EEG due to its relative non intrusiveness and portability—holds great potential for real world applications (reviewed in Abiri, Borhani, Sellers, Jiang, & Zhao, 2019; Belkacem, Jamil, Palmer, Ouhbi, & Chen, 2020; Carelli et al., 2017; Enriquez-Geppert, Huster, & Herrmann, 2017). For the case of episodic memory, if it is possible to detect the preparedness of the brain for learning with high accuracy, one could build a practical intervention system to support everyday learning. For instance, consider a student who wants to study for an exam. When their memory is working optimally, the system could give the student positive feedback that they are learning the material. However, once the system detects a decline in memory encoding, it could provide a warning to the student to take a break or study that material again, depending on the parameters that best facilitate learning. Such a system could also be beneficial for teachers who could decide when to give students a break or start new material. As a result, this hypothetical brain computer interface (BCI) system could prove helpful in customizing learning and memory for many individuals. BCI systems such as robotic exoskeletons and smart wheelchairs have been used to great success in the real-world for patients with motor impairments as these systems can detect the patients’ movement intentions with high accuracy (Belkacem et al., 2020). However, in the memory domain, this hypothetical system does not yet exist. Before such a system can be implemented in real life, it is first necessary to ensure that it is possible to accurately determine an individual’s episodic learning preparedness.

Machine learning methods are powerful tools that can make use of multidimensional data to successfully discriminate neural activity between different cognitive states. The standard machine learning procedure consists of feature extraction, feature selection or dimensionality reduction, and training a classifier (reviewed in Lotte et al., 2017). For feature extraction, some of the most common types of features used in EEG classification include voltage amplitude (Kaper, Meinicke, Grossekathoefer, Lingner, & Ritter, 2004), frequency power (Pfurtscheller, Neuper, Flotzinger, & Pregenzer, 1997), and phase of different frequency bands (Wei, Wang, Gao, & Gao, 2007). Statistical features including the mean and variance of the signal, and correlation between signals of two channels are also sometimes used, as are model-based or parametric features such as the coefficients derived from Autoregressive Modeling (Paranjape, Mahovsky, Benedicenti, & Koles’, 2001). Moreover, entropy-based features (Song & Liò, 2010) and common spatial pattern (CSP)-based features (Ramoser, Muller-Gerking, & Pfurtscheller, 2000) are frequently used in classification studies as well. A subset of features that are most informative for classifying brain states are selected using filter methods such as filtering by Fisher score (Abdi, 2006; McLachlan, 1992), and wrapper methods such as sequential forward selection (Dash & Liu, 1997). It is important to note that in some cognitive studies, this step is ignored, and all of the extracted features are used for classification. Lastly, the classifier is trained. Powerful classifiers that are commonly used in the literature include support vector machine (SVM) (Burges, 1998), linear discriminant analysis (LDA) (Fukunaga, 1990), logistic regression (Ng & Jordan, 2002), naïve bayes (Fukunaga, 1990), artificial neural networks (Bishop, 1995), and decision trees (A.K. Jain, R.P.W. Duin, & J. Mao, 2000).

There have been several attempts in the literature to separate single trial neural responses in EEG for events a person is likely to remember from those they are likely to forget using machine learning strategies (Astrand, 2018; Chakravarty, Chen, & Caplan, 2020; Ezzyat et al., 2018; Höhne, Jahanbekam, Bauckhage, Axmacher, & Fell, 2016; Noh, Herzmann, Curran, & de Sa, 2014; Noh, Liao, Mollison, Curran, & Sa, 2018). The most common features that were extracted in these studies included voltage and spectral power, while SVM, logistic regression, and LDA were the most common classifiers used. Performance varied across these studies but fell in the range of 59.6% to 69.2% accuracy. Most of these studies—and classification analyses in general—apply a particular methodology, for example logistic regression, power features, etc., without evaluating alternative approaches. Consequently, this raises a question about whether the set of features and classifiers chosen are optimally suited for classification of memory-related brain states. To address this question, some studies have compared different classification methods for distinct cognitive states in various domains including episodic memory (Arora et al., 2018) and motor imagery (Halme & Parkkonen, 2016; Höller et al., 2013; Tahernezhad-Javazm, Azimirad, & Shoaran, 2018). However, to the best of our knowledge, no study has directly and systematically compared different techniques at *every* step of classification (i.e., feature extraction, feature selection, classifier selection) of cognitive states in order to investigate which *set* of methods is optimal for a specific dataset.

In the current study, we systematically compared different options for every step of classification and their associated parameters to investigate which set of methods produces optimal performance for decoding successful versus unsuccessful episodic memory brain states. We used an EEG dataset that was collected during performance of an episodic memory task in adults across the lifespan (Mirjalili, Powell, Strunk, James, & Duarte, 2021). We used this dataset for two reasons. First, data were collected from young, middle-aged, and older adults which increases the generalizability of our results across different ages. Second, this is a rich dataset that enabled us to decode different memory states (i.e., item recognition, context recognition), address imbalance between classes (i.e., hits vs. misses), and solve binary and multiclass problems. Following the systematic comparison of different options for each stage of classification for this dataset, we applied the same procedures to three other datasets to assess the robustness and generalizability of our results.

A broader goal of this study was to enable researchers who are interested in performing robust and reliable single-trial classification analyses to make informed choices for the different stages of the analysis process. We believe that this study’s findings provide important insight into how the choice of extracted features, feature selection algorithm, and the classifier influence the reliability of predicting memory and other cognitive states. Importantly, while our analyses were conducted on an EEG dataset, most of the recommendations can also be used for performing classification on datasets that have been collected using other neuroimaging techniques such as fMRI. It is also essential to note that for each step of classification, while there are other techniques that have been used in machine learning research, we selected from those most commonly used in cognitive neuroscience research that are also available in analyses toolboxes.

## 2. Materials and Methods

### 2.1. Participants

The data included here were collected from individuals who participated in one or more previously published EEG studies in our lab (James, Strunk, Arndt, & Duarte, 2016; Mirjalili et al., 2021; Powell, Strunk, James, Polyn, & Duarte, 2018; Strunk, James, Arndt, & Duarte, 2017). The participants consisted of 65 right-handed adults (26 women), ages 18–74. All subjects were native English speakers and had normal or corrected vision. Participants were compensated with course credit or $10/hour and were recruited from the Georgia Institute of Technology and surrounding community. None of the participants reported any psychiatric or neurological disorders, vascular disease, or using any medications that affect the central nervous system. Participants completed a standardized neurological battery of neuropsychological tests which consists of subtests from the memory assessment scale (Williams, 1991), including list learning, verbal span forward and backwards, recognition, visual recognition, immediate and delayed recall, recall, reproduction, and delayed recognition. Participants were excluded if their scores were above or below two standard deviations of the group mean. Furthermore, older adults were administered the Montreal Cognitive Assessment (MoCA) (Nasreddine et al., 2005) to test further for mild cognitive impairments. Participants scoring less than 26 on the MoCA were excluded. All participants signed consent forms approved by the Georgia Institute of Technology Institutional Review Board. Five older participants (61–76 years) were excluded in this study: two for noisy EEG (i.e., DC drift, movement), two for not understanding the task procedures, and one for computer malfunction.

### 2.2. Stimuli

Four hundred thirty-two grayscale images of objects were chosen from the Hemera Technologies Photo-Object DVDs and Google images. During encoding, 288 of these objects were presented. Each grayscale object was presented on the center of the screen with white background. Scenes and color squares were presented to the left or right of the object. The locations of the context features (i.e., color or scene) were counterbalanced across blocks so that they were shown an equal number of times on the left and right across subjects. The scenes included color photos of a studio apartment, cityscape, or island. The colored squares were green, brown, or red. Each of the context and object pictures spanned a maximum vertical and horizontal visual angle of approximately 3°. At retrieval, all 288 objects were included in the memory test as well as 144 new object images that were not shown during encoding. Study and test items were counterbalanced across subjects.

### 2.3. Experimental task

**Figure 1.**
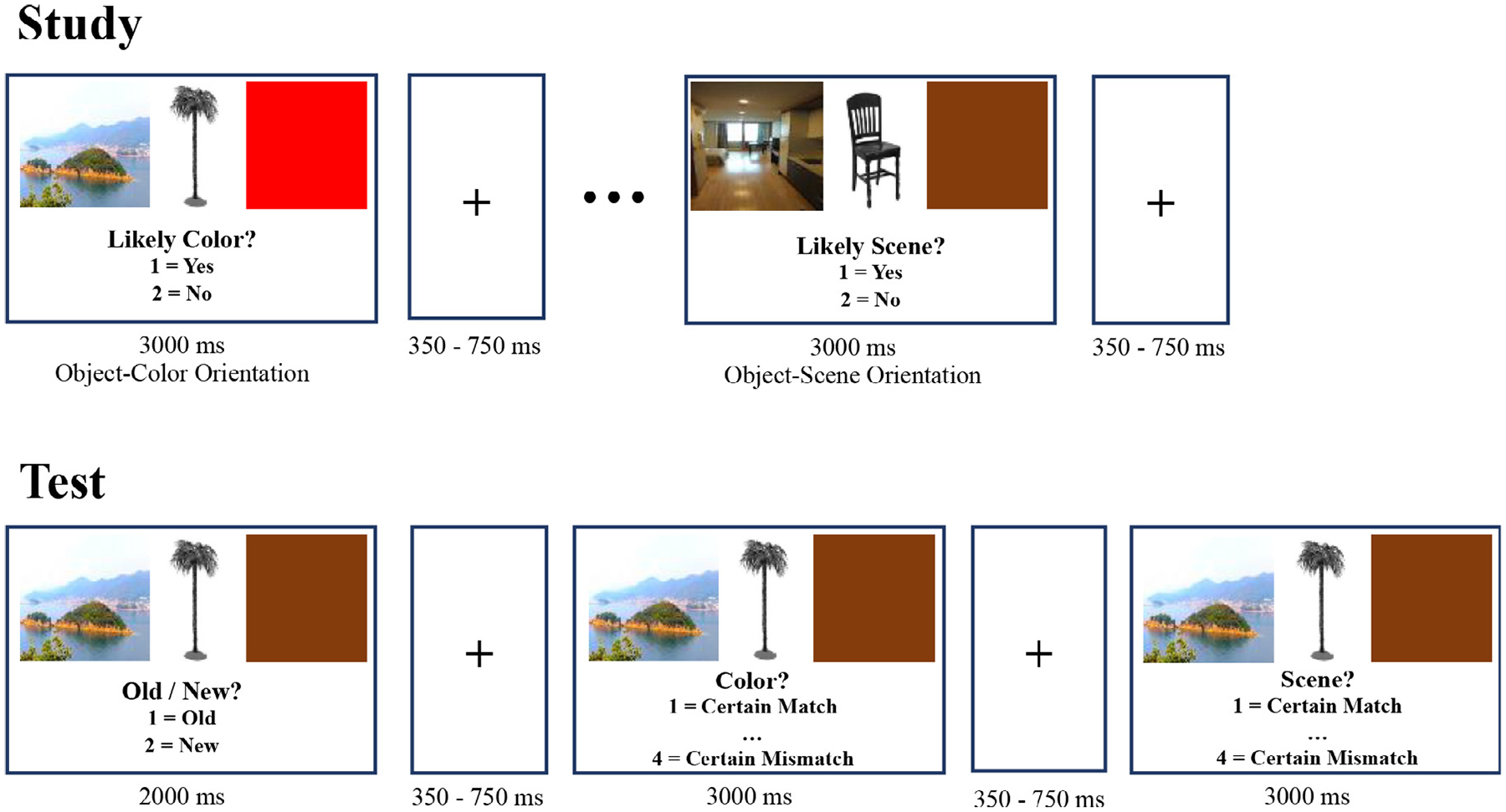
Task design for the episodic memory study. The scenes in this figure are taken from Creative Commons.

Figure 1 shows the procedure used at the study and test stages. Prior to the beginning of each stage, participants were provided instructions and given 10 trials for practicing. For each encoding trial, participants were instructed to pay attention to either the scene or colored square, which served as the target context for that trial. For the study stage, participants were asked to make a subjective yes/no assessment about the relationship between the object and either the colored square (i.e., is this color likely for this object?) or the scene (i.e., is this object likely to appear in this scene?).

Within the study phase there were four blocks where each block included four mini-blocks, each of which consisted of 18 trials. Before beginning each mini-block, participants were provided a prompt (e.g., “Now you will assess how likely the color is for the object” or “Now you will assess how likely the scene is for the object”). Since prior evidence has found that memory performance in older adults is more disrupted when they have to switch between two different types of tasks (Kray & Lindenberger, 2000), mini-blocks were used to orient the participant to which context they should focus on in the upcoming trials. In addition, each trial in a mini block had a reminder prompt shown underneath the pictures during study trials (see Figure 1).

At the test stage, participants were presented with both old and new objects. Similar to the study phase, each object was presented by both a colored square and a scene. For each object, the participant initially decided whether it was an old or a new image. If the participant detected the object as a new one, the next trial began after 2000 ms. If participants stated that it was old, then they were asked to make two additional judgments about each context feature and determine their certainty about their assessment (i.e., one about the colored square and another about the scene). The order of the second and third questions was counterbalanced across participants. For old items, the pairing was set so that an equal number of old objects were presented with: (1) both context images matching those presented at encoding stage, (2) only the scene matching, (3) only the color matching, and (4) neither context images matching. Responses to the context questions were made on a scale from 1 (certain match) to 4 (certain mismatch). In total, there were four study and four test blocks. Young adults finished all four study blocks before the four test blocks. For older adults aged 60 and up, to better equate item memory performance with young adults, the memory load was halved so that they finished a two- block study-test cycle twice (two study, two test, two study, two test). Both younger and older adults finished a short practice of both the study and test blocks prior to beginning the first study block. Consequently, both younger and older adults knew of the following memory test.

### 2.4. EEG recording

Continuous scalp-recorded EEG data was recorded from 32 Ag-AgCl electrodes using an ActiveTwo amplifier system (BioSemi, Amsterdam, Netherlands). Electrode position is based on the extended 10–20 system (Nuwer et al., 1998). Electrode positions included: AF3, AF4, FC1, FC2, FC5, FC6, FP1, FP2, F7, F3, Fz, F4, F8, C3, Cz, C4, CP1, CP2, CP5, CP6, P7, PO3, PO4, P3, Pz, P4, P8, T7, T8, O1, Oz, and O2. External left and right mastoid electrodes were used for referencing offline. Two additional electrodes recorded horizontal electrooculogram (HEOG) at the lateral canthi of the left and right eyes and two electrodes placed superior and inferior to the right eye recorded vertical electrooculogram (VEOG). The sampling rate of EEG was 1024 Hz with 24-bit resolution without high or low pass filtering.

### 2.5. EEG preprocessing

Offline analysis of the EEG data was conducted in MATLAB 2015b using the EEGLAB (Delorme & Makeig, 2004), ERPLAB (Lopez-Calderon & Luck, 2014), and FIELDTRIP (Oostenveld, Fries, Maris, & Schoffelen, 2011) toolboxes. The continuous data were down sampled to 256 Hz, referenced to the average of the left and right mastoid electrodes, and band pass filtered between 0.5 Hz and 125 Hz. The data were then epoched from −1000 ms prior to stimulus onset to 3000 ms. The time range of interest was −300 ms to 2000 ms, but a longer time interval is needed to account for signal loss at both ends of the epoch during wavelet transformation (i.e., edge effects). Each epoch was baseline corrected to the average of the whole epoch, and an automatic rejection process removed epochs in which a blink occurred during stimulus onset or epochs with extreme voltage shifts that spanned across two or more electrodes. The automated rejection processes identified epochs with the following parameters in the raw data: 1) The voltage range was higher than 99th percentile of all epoch voltage ranges within a 400 ms time interval (shifting in 100 ms intervals at each epoch). 2) The linear trend slope was larger than the 95th percentile of all epoch ranges with a minimum R2 value of 0.3. 3) The voltage range was greater than 95th percentile of all epoch voltage ranges within a 100 ms time interval (shifting in 25 ms intervals across each epoch), between −150 and 150 ms from stimulus onset for frontal and eye electrodes only. Subsequently, an independent component analysis (ICA) was run on all head electrodes to identify ocular artifacts (i.e., blinks and horizontal eye movements). Components related to ocular artifacts were removed from the data by visually scrutinizing the topographic component maps and component time course with the ocular electrodes. 32 ICA components were computed and the mean of the number of components removed was 2.43 (SD = 0.722). Each epoch was re-baselined to the −300 to −100 ms time period before stimulus onset since the epochs were no longer baselined to a specific time period after omitting components related to ocular activity. This was done solely for the purposes of visual inspection and identification of additional artifacts in each epoch (e.g., amplifier saturation, spiking, extreme values, uncorrected ocular activity), and does not affect the frequency decomposition. If a dataset had a noisy electrode (e.g., higher than 30% of the data required to be rejected), it was removed from the processing stream and interpolated using the nearby channels to estimate the activity within the bad channel before running the time frequency procedure (Delorme & Makeig, 2004). After all processing stages, about 13% (range of 2.1% to 42.7%, SD = 8%) of the epochs were removed on average across participants.

**Figure 2.**
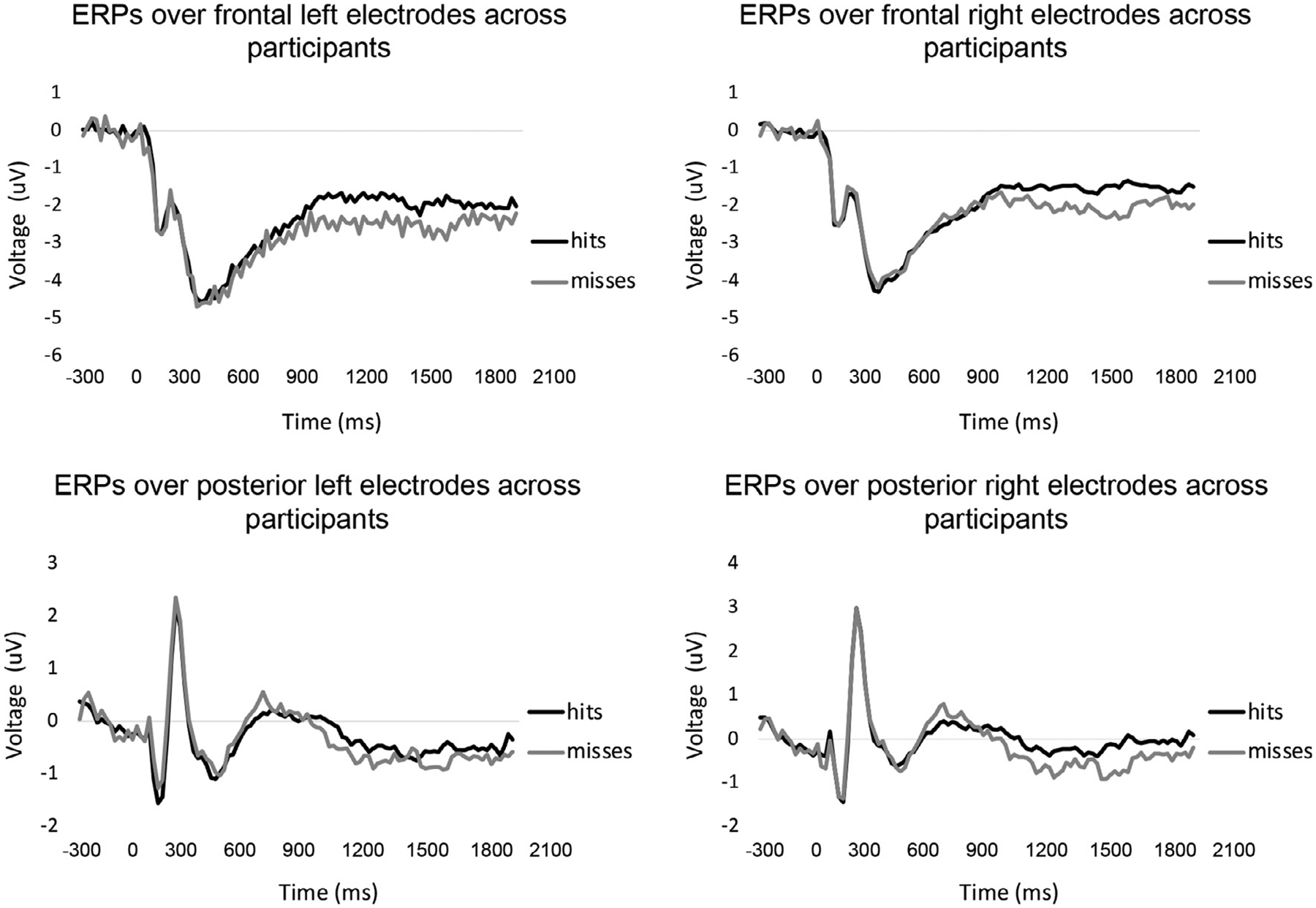
The grand averages of hits and misses across participants over the four electrode regions including frontal right, frontal left, posterior left, and posterior right.

Each epoch was transformed into a time frequency representation by Morlet wavelets (Percival, Walden, & others, 1993) with 78 linearly spaced frequencies from 3 to 80 Hz, at 5 cycles. During the wavelet transformation, each epoch was decreased to the time interval of interest and down sampled to 50.25 Hz (Cohen, 2014). The average number of trials for encoding was 250 (range 181:274, SD = 23.78). For visualization of the data, we show some example grand averages in Figure 2. Specifically, for each participant, we averaged the voltage across the electrodes of frontal right, frontal left, posterior left, and posterior right for item hits and misses separately, and computed grand averages across subjects.

### 2.6. Summary of the cognitive problems that were investigated in this study

**Figure 3.**
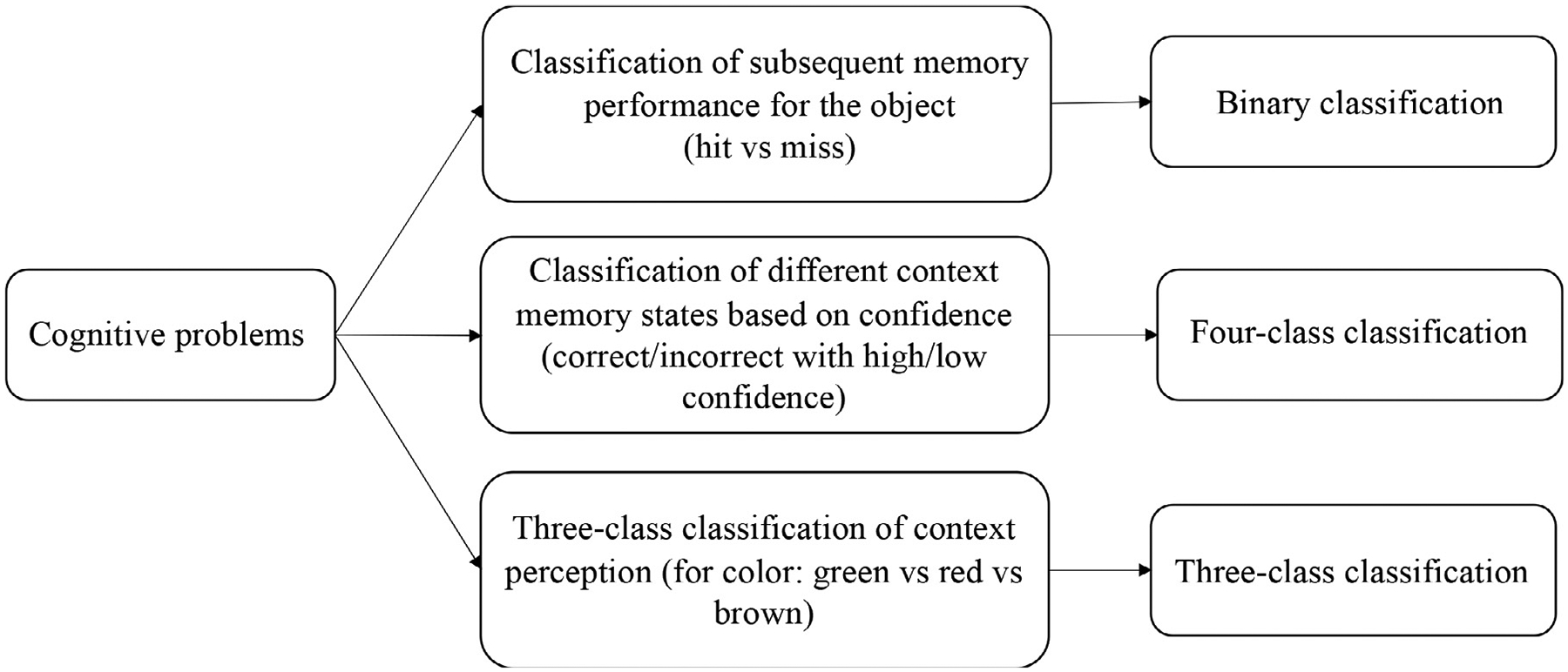
Summary of cognitive problems to be assessed in this study.

Figure 3 demonstrates a summary of cognitive problems for which we performed classification analyses. Although EEG data were recorded during both the encoding and retrieval phase of the experiment, we performed the analyses for the encoding period only. The reason being that an interesting potential future application of this approach is to classify optimal brain states for learning in real time, as discussed in the introduction. Furthermore, the goal of this study was not to classify encoding and retrieval brain states per se, but to systematically compare different types of extracted features, feature selection methods, and choices of classifier for the classification of memory states in the same dataset. Importantly, we only performed classification analysis if all of the classes contained at least 20 trials for each participant.

In the first analysis, we distinguished the trials where the object was later remembered from those where the object was later forgotten. In the second analysis, we performed a four-class classification between correct and incorrect color context memory trials associated with high or low confidence decisions. Third, we performed three-class classification between perception of the three different colors^1^. We performed these analyses since they allowed us to explore the efficiency of the methods for various types of problems in terms of the cognitive states of interest, the degree of imbalance between classes, and whether the classification problem was a binary or a multi-class problem. All of the classification analyses were conducted in MATLAB 2018a on an Apple MacBook Pro (operating system: macOS Big Sur 11.5.2 (20G95), processor: 2.3 GHz Dual-Core Intel Core i5, RAM: 8 GB 2133 MHz LPDDR3).

### 2.7. Feature extraction

As discussed previously, each epoch of the EEG signals was transformed into time-frequency representations using Morlet wavelets. Our analysis was based on the time series of power values for each frequency band, epoch, and electrode. Numerically, for each trial there is a matrix that has the same number of rows as the number of electrodes (*N*) and same number of columns as the number of power samples across the time (*T*). Importantly, for each frequency band, we normalized the power values within all electrodes and time points across all the trials for each participant so that the power values have similar ranges across different trials and electrodes (A.K. Jain et al., 2000). We found in piloting that normalization of power values reduced the running time, especially for logistic regression and SVM. We extracted 6768 features using five types of features in this study. These features are extracted from the normalized total power—sum of powers within the specific frequency band—of different frequency bands including theta (3–7 Hz), alpha (8–12 Hz), beta (13–30 Hz), and gamma (35–80 Hz). Specific details of the features are described below.

#### 2.7.1. Statistical features

These features include statistical mean, variance, and correlation between signals. To be more specific, each trial has a matrix of *N* × *T* power values for *N* electrodes and *T* time samples. In this study, for each electrode, we divided the T power samples into nine 400 millisecond time windows with consecutive time windows overlapping for 200 ms (i.e., [0 400], [200 600], [400 800], …, [1600 2000] ms) in order to reduce processing time (see supplementary material for “The importance of temporal resolution of extracted features”). The 400 ms window is similar to ones used in previous studies using similar memory designs that showed subsequent memory effects spanning 200 ms or longer (Morton et al., 2012; Morton & Polyn, 2017). Each 400 ms time window consisted of 20 time samples. In addition, while EEG signal has a non-stationary nature (i.e., its statistical characteristics change over time), it behaves closer to stationary in the short time windows; hence, the extracted features from shorter time windows are more reliable and useful for classification. For each electrode and time window, the mean and the variance of the 20 power values across the 20 time samples within that 400 ms time window for each frequency band were extracted. Furthermore, in order to be computationally practical, for correlations, we divided the 32 scalp electrodes into four regions, namely frontal right, frontal left, posterior right, and posterior left regions. We extracted average power for each frequency band across the electrodes for each region. For each pair of regions of electrodes of single-trials, Pearson correlation between average power samples from the same time window and frequency bands were computed (e.g., correlation between time series of average theta power at left frontal with the right posterior region of electrodes in the 400–800ms time window).

#### 2.7.2. Features based on entropy

Entropy is a measurement of signal’s uncertainty. There are several choices for definition of entropy, but we used Shannon entropy, which in the discrete form is defined as (Shannon, 1948):

$$H(x)=-\underset{x}{\sum }p(x){\text{log}}_{2}(p(x))$$

where *p*(*x*) denotes the probability density that *x* occurs. In this study, for each electrode and time window, Shannon entropy of the power values was computed for each frequency band.

#### 2.7.3. Phase-based features

The instantaneous phase for each time window and frequency band was estimated using the Hilbert Transform. The phase is computed by $(t)=tan^{-1}\left(\frac{\text{imaginar }y(t)}{\text{real}(t)}\right)$. To obtain the mean phase for each time window and frequency band for each electrode, we projected the phase values across the time window, wrapped them to the [0 2*π*) range of phases, onto the unit circle in the complex plane, and then computed the absolute value of the mean phase. Moreover, to calculate the mean phase synchronization between each pair of electrode regions within each time window and within each frequency band, we first calculated the mean phase for each of the two electrode regions in that pair. Subsequently, at each of the time samples within the 400 ms time window, we projected the phase differences between the two electrode regions, wrapped them to the [0 2*π*) range of phases, onto the unit circle in the complex plane. Next, we computed the absolute value of the mean phase difference between the two signals (Kreuz, 2011; Mardia, 1975; Mormann, Lehnertz, David, & E. Elger, 2000).

#### 2.7.4. Model-based features

Estimated parameters of parametric models can be used as features in classification problems (Pardey, Roberts, & Tarassenko, 1996). We used the commonly used Autoregressive model (AR) (Lotte et al., 2017). In the AR model, the power of the signal at each timepoint is considered as a linear combination of the power values of the signal at *p* previous timepoints, in addition to white noise

$$x(n)=\overset{p}{\underset{i=1}{\sum }}\alpha_{i}x[n-i]+u[n]$$

Where each *α*_*i*_ is one of the coefficients or parameters of the model that needs to be estimated according to the observed signal. We used *p* = 4 which is a common value used in the EEG literature (Pardey et al., 1996; Sardouie & Shamsollahi, 2012). Subsequently, for each region of electrodes and time window, an AR model was fitted to the average time series of power values for each frequency band. The obtained model parameters were used as features.

#### 2.7.5. Features based on Common Spatial Pattern (CSP)

The CSP algorithm tries to increase the separability between classes by learning spatial filters which maximize the power of the filtered signal for one class and minimize the power for the other class (Ramoser et al., 2000). The average covariance matrices of the trials of each class are calculated, creating $\bar{C_{1}}$ and $\bar{C_{2}}$ for the two classes. Using the concept of eigenvalue decomposition, an optimization problem of $w=\text{arg }\underset{a}{\text{max}}\frac{w^{T}\bar{C_{1}}w}{w^{T}\bar{C_{2}}w}$ is solved to obtain the optimal spatial filters. The spatial filters optimally project the signals into a new space in which the signal at each projected electrode is a linear combination of the signals across all original electrodes.

In this study, for each frequency band and time window, CSP filters were computed and then applied to the time series of power values across all electrodes. The variances of the filtered signals at each projected electrode were used as features.

### 2.8. Feature Selection

After features have been extracted, a subset can be selected for classification. This step is often skipped in cognitive neuroscience studies. However, it is possible that some useful information will be missed, and classification performance will be suboptimal. Moreover, the machine learning literature strongly supports feature selection for improving classification performance and avoiding overfitting (Dias, Jacinto, Mendes, & Correia, 2009; Koprinska, 2009; Lotte et al., 2017). There are a few approaches to select the best features, but we focus on two of these strategies: filter and wrapper methods, and their combination. We compare these selection methods against no feature selection.

#### 2.8.1. Filter Methods

For filter methods, evaluation of features is independent of the classification algorithm. Features are evaluated based on their information content, such as interclass distance, information-theoretic measures, etc. We focused on Fisher’s criterion since it is one of the most common approaches (Gu, Li, & Han, 2011). The Fisher score for the feature *f* can be computed using the following formula:

$$\text{score}(f)=\frac{{\left(\mu_{1}-\mu \right)}^{2}+{\left(\mu_{2}-\mu \right)}^{2}}{\sigma_{1}^{2}+\sigma_{2}^{2}}$$

where *μ*_1_ and $\sigma_{1}^{2}$ represent the average and variance of *f* for the trials that belong to Class 1, *μ*_2_ and $\sigma_{2}^{2}$ represent the average and variance of *f* for the trials that belong to Class 2, and *μ* represent the average of *f* for all trials. Features are then sorted by their scores, and those with highest scores are selected. Regarding the number of features, there is no required number. However, a rule of thumb is to use the square root of the number of available trials (Hua, Xiong, Lowey, Suh, & Dougherty, 2004). In this study, to be consistent across all analyses and participants, we selected the 10 features with the highest Fisher scores.

#### 2.8.2. Wrapper Methods

While the evaluation of individual features is independent of the classifier for filter methods, wrapper methods use a pattern classifier that appraises feature subsets by their predictive accuracy (rate of recognition on test data) using cross-validation or statistical resampling (Guyon & Elisseeff, 2003). On approach for selecting the optimal subset of features is to exhaustively search through all subsets and pick the best performing. However, the number of possible subsets makes exhaustive search computationally expensive and impractical. One of the most commonly used strategies to overcome this issue, which we applied here, is to apply sequential forward selection (SFS) (Guyon & Elisseeff, 2003; Mitchell, 1997). SFS chooses the best single feature according to a specific criterion function (five-fold cross validation performance in this study). Next, pairs of features and then triplets are chosen from the remaining feature set, and this process continues until a subset of predefined number of features are chosen.

#### 2.8.3. Combination of Filter and Wrapper Methods

SFS methods alone can be impractical when there are many features to search through. A much faster approach is to evaluate the features initially using the filter method, then search through only those that have received high scores (Arbabi, Shamsollahi, & Sameni, 2005; Chuang, Ke, & Yang, 2008; Hameed, Petinrin, Hashi, & Saeed, 2018; Maldonado, Weber, & Famili, 2014). It is important to note that the wrapper only considers the Fisher score of the features, and it is not provided with any other information about the features (i.e., time windows, types, frequencies, electrodes) to identify the best features. In this study, we compared the filter approach alone with the combination of filter and wrapper methods and compared both to no feature selection. To be consistent across all analyses and participants, and to avoid the risk of overfitting and underfitting based on the number of trials, the wrapper searched for the best 10 features (~4% of the trials) among the 100 features (~40% of the trials) that had the highest Fisher scores (See supplementary material for “Impact of the number of features”).

### 2.9. Training a Classifier

There are many classifiers that are used in the literature, but we used four of the most commonly used classifiers including SVM, logistic regression, LASSO, and naïve Bayes. Importantly, for many cognitive problems, there are two classes (e.g., remembered vs. forgotten, move left vs. move right) and many classifiers are either specialized or well suited for binary classification but can be generalized to perform multiclass classification. Additionally, each of these classifiers has advantages and disadvantages that we describe below.

Before delving into the descriptions of the classifiers, it is important to note that in many classification analyses, the classes have different numbers of trials, and the classifier will have a skewed tendency to generate test labels as the class with the majority of the trials. Handling the imbalance issue can be done by either under-sampling the class with more trials or over-sampling the class with fewer trials. Since the number of trials in our data is relatively low, particularly for forgotten trials, as in most memory studies, we did not use under-sampling as it would ignore some information that could be helpful for classification (see supplementary material for “Different methods for handling class imbalance”). Instead, we used the synthetic minority oversampling technique or “SMOTE” to generate new data points by over-sampling the minority class to create synthetic trials (Arora et al., 2018; Chakravarty et al., 2020; Chawla, Bowyer, Hall, & Kegelmeyer, 2002). Minority trials were created until both classes had the same number of trials for training.

#### 2.9.1. Support Vector Machine (SVM)

SVM tries to separate the trials of each class using a linear hyperplane (Burges, 1998). The linear hyperplane should be specified in a way that maximizes the distance from the hyperplane to the closest trial from each class. Suppose the equation for optimal hyper plane is written as:

$$w^{T}x+b=0$$

where *x* is a set of points, *w* is a vector to the hyperplane, and *b* is the offset term. It can be shown that the margin width is equal to:

$$M=\frac{2}{\sqrt{w.w}}=2/w$$

SVM performs well in higher dimensions (i.e., the number of input features is relatively high, and it has low risk of overfitting). However, it is slow for larger datasets and does not perform well when the data are especially noisy (Roy, Kar, & Das, 2015; Uddin, Khan, Hossain, & Moni, 2019; Van Messem, 2020).

#### 2.9.2. Logistic regression

Logistic regression is a modified version of the linear regression model that is used for binary classification problems. It squeezes the output of a linear equation between 0 and 1 by using the logistic function is defined as:

$$\text{logistic}(x)=\frac{1}{1+e^{-x}}$$

Logistic regression has low risk of overfitting, is simple to implement, its outputs have probabilistic interpretations, it can be used for both binary and multiclass classification problems, and it can be updated easily after adding new data. Nonetheless, it does not perform well on non-linear scenarios, requires more data to achieve stability, and is not flexible enough to capture more complicated non-linear relationships between the output and the inputs and underperforms when the decision boundary is not linear (Chang, 2020; Holdnack, Millis, Larrabee, & Iverson, 2013; Uddin et al., 2019).

#### 2.9.3. LASSO

LASSO is a type of regression that tries to define the output (the class) based on a linear combination of the inputs (Tibshirani, 1996):

$$\underset{\beta }{\text{min}}\|y-X\beta {\|}^{2}+\lambda \|\beta {\|}_{1}$$

where *y* is the output class and the classes are treated as real numbers, *X* is the input vector of the selected features, and ∥*β*∥_1_ denotes the nonzero coefficients for the linear regression. As a result, the goal is to minimize the mean squared error of the output estimation while limiting the number of features that will be used for the output estimation by having non-zero weights in the regression. LASSO has low risk of overfitting and can be updated easily after adding new data. If features are correlated, LASSO arbitrarily choses one and ignores the others. Moreover, it is not flexible enough to capture more complicated non-linear relationships between the output and the inputs (Melkumova & Shatskikh, 2017; Pereira, Basto, & da Silva, 2016).

#### 2.9.4. Naïve Bayes

This classifier depends on the conditional probability of the occurrence of events from each class (Fukunaga, 1990). In mathematical terms:

$$\{\begin{array}{l} if\;p\left(x|C_{1}\right)>p\left(x|C_{2}\right)\;\text{then }x\in C_{1}\\ if\;p\left(x|C_{1}\right)<p\left(x|C_{2}\right)\;\text{then }x\in C_{2} \end{array}$$

where *x* is a data point, *C*_1_ and *C*_2_ represent the classes 1 and 2, and *p* indicates the probability.

For classification, an estimation of the probability distribution of each class is needed. However, since no information about the true probability distribution and its parameters is known, it is assumed that it follows a normal distribution. By assuming a normal distribution, the following parameters for class *i* are defined:

$$A_{i}=-0.5\times \Sigma_{i}^{-1}$$

$$b_{i}=\Sigma_{i}^{-1}\times \mu_{i}$$

$$c_{i}=-0.5\times \mu_{i}^{T}\times \Sigma_{i}^{-1}\times \mu_{i}-0.5\times \text{log }\left|\Sigma_{i}\right|+\text{log }\left(P_{i}\right)$$

$$d_{i}(x)=x^{T}\times A_{i}\times x+b_{i}^{T}\times x+c_{i}$$

where Σ_i_ and *μ*_*i*_ are the covariance matrix and the mean vector of the data points that belong to class *i* and $\Sigma_{\text{i}}^{-1}$ is the inverse matrix of Σ_i_. By the above definitions, it can be inferred that:

$$\{\begin{array}{l} if\;d_{1}(x)>d_{2}(x)\;\text{then }x\in C_{1}\\ if\;d_{1}(x)<d_{2}(x)\;\text{then }x\in C_{2} \end{array}$$

Naïve Bayes is easy and quick to implement, is insensitive to irrelevant features, does not require a large amount of training data, and works on non-linear problems. On the other hand, it is not a good estimator of the probabilities, it does not improve the posterior probabilities iteratively, presence of dependency between features negatively impacts its performance, and it assumes the features follow a normal distribution which is not necessarily true and can affect its performance (Narisetty, 2020; Rice, 2014; Uddin et al., 2019; Yeturu, 2020). However, this was not a problem in our dataset as the features followed a normal distribution.

### 2.10. Multiclass Classification and the Generalization Methods

There are many techniques for solving multiclass classification problems. We used two of the most commonly used methods.

#### 2.10.1. Classification Based on the Voting Method

This strategy is a generalization of binary classification to a multiclass classification problem. In any classification analysis, besides assigning labels, the classifier gives a score for each trial indicating how certain the classifier is about the associated label. In this study, for the voting method, a one against others approach is used. First, the trials of the second and third class are combined to create a new merged class, turning the multiclass classification into a binary classification problem (i.e., Class 1 vs. Classes 2/3). The same process is repeated for Class 2 against Classes 1/3, and so on. In this example, each trial receives three labels and three sets of scores, and the class to which the highest sum of scores is associated overall will be selected as the final label of that trial. Importantly, this method works regardless of the number of classes in the multiclass problem. Another common voting method variation is to use a one against one approach (Aly, 2005; Bishop, 1995; Chmielnicki & St ąpor, 2016; Hsu & Lin, 2002). For an n-class problem (*n* > 3), it is a more time-consuming approach. However, we compared this approach to the one vs. others and performance was similar (see supplementary material: “Different voting method approached for multiclass classification”).

#### 2.10.2. Classification Using Binary Decision Trees

While the voting method uses the scores of the classification output and maintains all of the candidate labels throughout generalization, the binary decision tree method reduces the candidate labels throughout generalization. To elaborate, a binary classification is performed at each node of the tree. At each new node, the groups (if they consist of more than one class) are partitioned into two subgroups and the classification is performed again. The same process is repeated until each leaf of the tree represents a single class. While the partitioning can be done arbitrarily at each node of the tree, we used an algorithm that first performs binary classification on each pair of classes and then outputs the partition with the highest performance (Mirjalili, Sardouie, & Samiee, 2019). We used this approach in this study. This algorithm can be used for any multiclass problem regardless of how many classes they have.

##### Code and data accessibility

The custom code that we used in this study, the data and results that support the findings of this study are available from https://doi.org/10.17605/OSF.IO/GYFPA. Moreover, the EEG and MEG working memory and motor imagery datasets that we used for assessing the generalizability of the methods are available from Collaborative Research in Computational Neuroscience (CRCNS) data sharing (http://crcns.org/data-sets/pfc/pfc-5/about-pfc-5), Human Connectome Project (https://www.humanconnectome.org/study/hcp-young-adult/document/500-subjects-data-release), and Berlin Brain-Computer Interface websites (http://www.bbci.de/competition/iv/desc_1.html) respectively.

**Figure 4.**
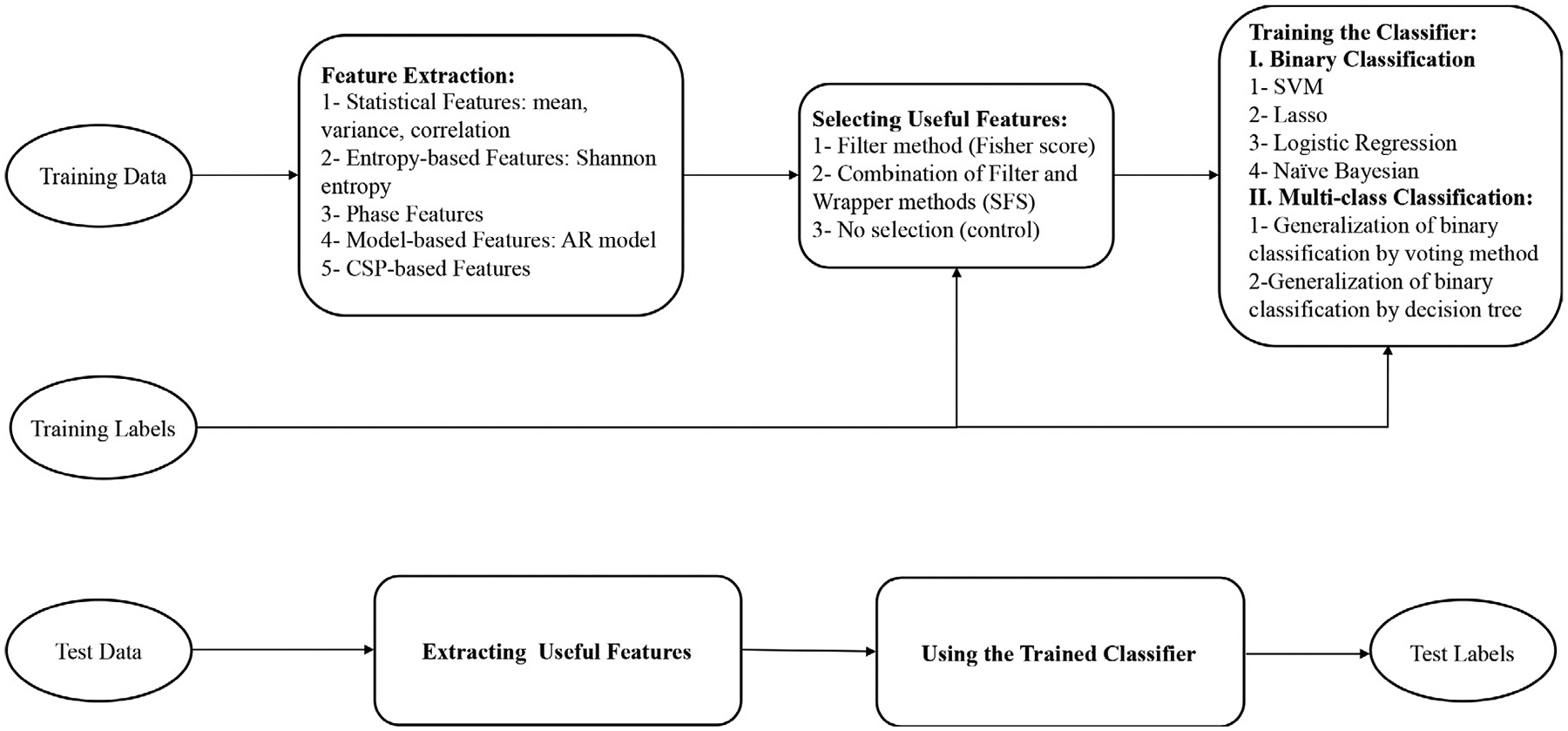
A summary of the used methodology

To summarize, for each time segment, frequency band, and electrode we extracted several types of features: mean power, mean variance, mean entropy, and mean instantaneous phase. Phase synchronies, correlations between the 4 regions of electrodes, AR coefficients at each of the 4 regions, and CSP-based features—32 variances at 32 electrodes after applying the spatial filters, for 6768 total features. A summary of the methods that we used are shown in Figure 4.

## 3. Results

### 3.1. Binary Classification

#### 3.1.1. Binary Classification of Subsequent Item Memory Performance

**Figure 5.**
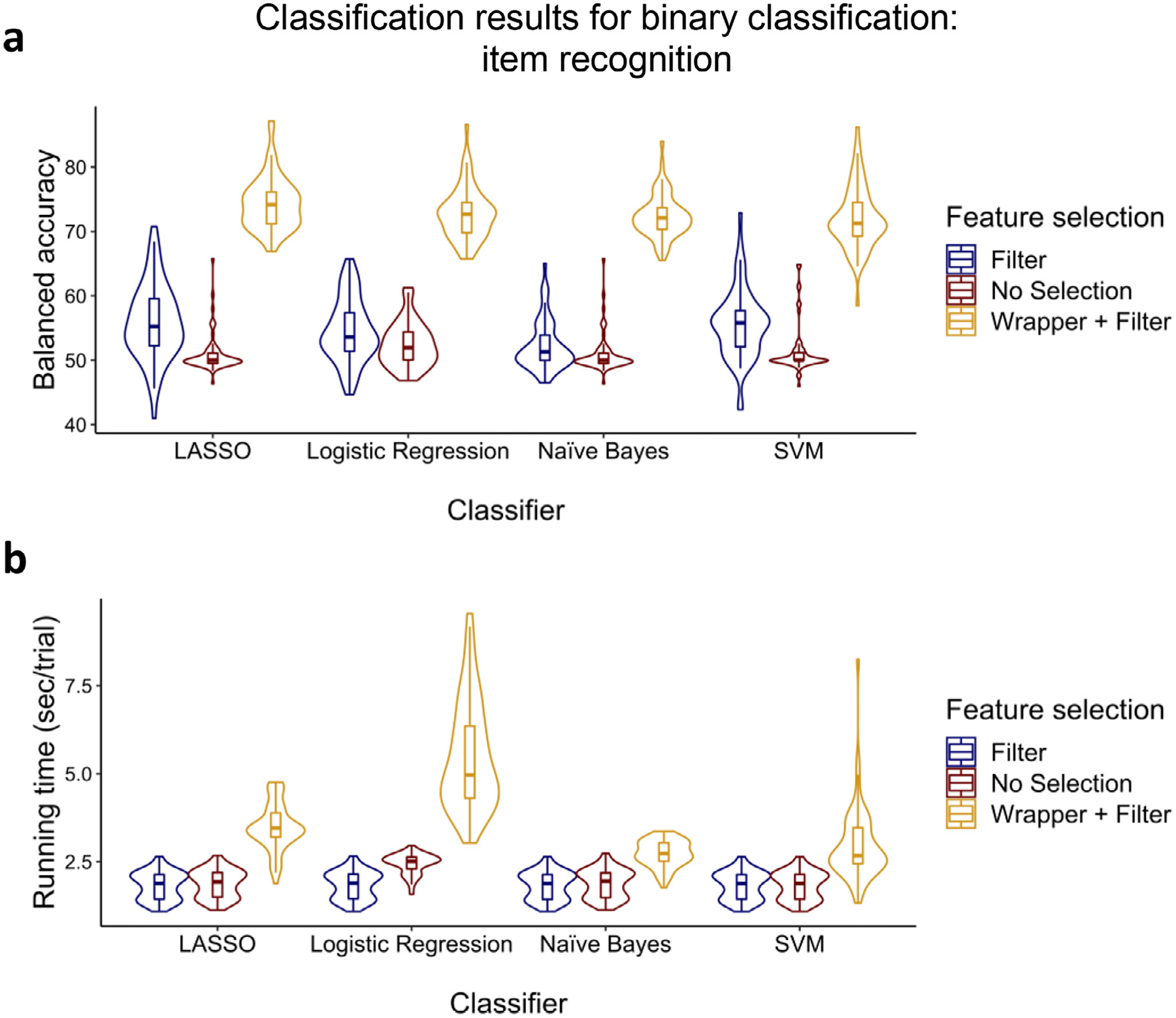
Comparisons of balanced accuracy and running time for different feature selection methods and classifiers for item recognition (hits vs. misses). **A)** balanced accuracy; and **B)** running time (in seconds per trial). The violin plots indicate the distribution of data scores. The box plots are shown inside the violin plots. The average chance levels—the average of the 95^th^ percentile of the balanced accuracy values in the null distribution—across participants were in the range of 50.2 to 53.1% for all analyses. It should be noted that classification performances were significantly above the empirical chance levels across the participants for these analyses.

In this problem, we were interested in classifying the trials at encoding based on the subsequent memory for the objects (i.e., item hits vs item misses). The summary of the results can be found in Figure 5.

To statistically compare feature selection methods and classifiers, we ran a Feature selection (filter, filter + wrapper, no selection) × Classifier (LASSO, logistic regression, naïve Bayes, and SVM) ANCOVA for both balanced accuracy (see supplementary material: “Measures Used to Assess a Classifier’s Performance”) and running time with age as a covariate. For balanced accuracy, significant main effects of Feature selection [*F*(2, 707) = 1802.02, *p* < 0.001, $\eta_{p}^{2}=0.836$] and Classifier [*F*(3, 707) = 6.53, *p* < 0.001, $\eta_{p}^{2}=0.027$] and the interaction [*F*(6, 707) = 4.01, *p* < 0.001, $\eta_{p}^{2}=0.033$] were observed, after controlling for Age, which was not a significant predictor [*F*(1, 707) = 2.33, *p* = 0.127, $\eta_{p}^{2}=0.003$], Follow-up t-tests showed that the filter + wrapper selection method outperformed the other two selection methods [all *t*s > 19.54, *p*s < 0.001], and filter alone outperformed no selection [all *t*s > 2.16, *p*s < 0.018] for all classifiers. Moreover, as can be seen in Figure 5a, LASSO outperformed others for the filter + wrapper method [all *t*s > 3.15, *p*s < 0.002]. For the filter method, LASSO outperformed others [all *t*s > 2.13, *p*s < 0.019] except SVM [*t*(59) = 0.56, *p* = 0.288]. Lastly, for no selection, logistic regression outperformed others [all *t*s > 4.46, *p*s < 0.007] except SVM [*t*(59) = 1.60, *p* = 0.058].

For running time, main effects of Feature selection [*F*(2, 707) = 243.52, *p* < 0.001, $\eta_{p}^{2}=0.408$] and Classifier [*F*(3, 707) = 51.27, *p* < 0.001, $\eta_{p}^{2}=0.179$] and the interaction [*F*(6, 707) = 29.13, *p* < 0.001, $\eta_{p}^{2}=0.198$] were all significant after controlling for Age, which was not a significant predictor [*F*(1, 707) = 3.22, *p* = 0.073, $\eta_{p}^{2}=0.005$], Follow-up t-tests confirmed that the filter selection method was faster than the other two methods [all *t*s > 7.33, *p*s < 0.001] while no selection was faster than the filter + wrapper [all *t*s > 7.99, *p*s < 0.001] for all classifiers. As can be seen in Figure 5b, naïve Bayes was faster than the others for the filter + wrapper method [all *t*s > 1.75, *p*s < 0.043]. For the filter method, SVM was faster than the others [all *t*s > 4.50, *p*s < 0.001] except naïve Bayes [*t*(59) = 1.29, *p* = 0.101]. For no selection, SVM was faster than the others [all *t*s > 8.88, *p*s < 0.001].

Lastly, as predicted, after controlling for age, classifier performance was positively predictive of item d-prime [*ρ*(57) = 0.345, *p* = 0.007]. For this analysis, we computed the partial Pearson correlation between item d-prime and the highest-performing classification method (using LASSO when the effective features are selected using the filter + wrapper method) while controlling for age.

### 3.2. Multiclass Classification

#### 3.2.1. Four-Class Classification of Subsequent Context Memory Performance

We used two approaches to generalize a binary classification problem into a multiclass problem—namely the voting method and the binary decision trees. Since the previous results showed that selecting the effective features using the combination of filter and wrapper methods led to the highest performances, we used that approach for this problem (see supplementary material for “Comparison of feature selection methods in multiclass classification”). We then used both generalization strategies for all four classifiers.

**Figure 6.**
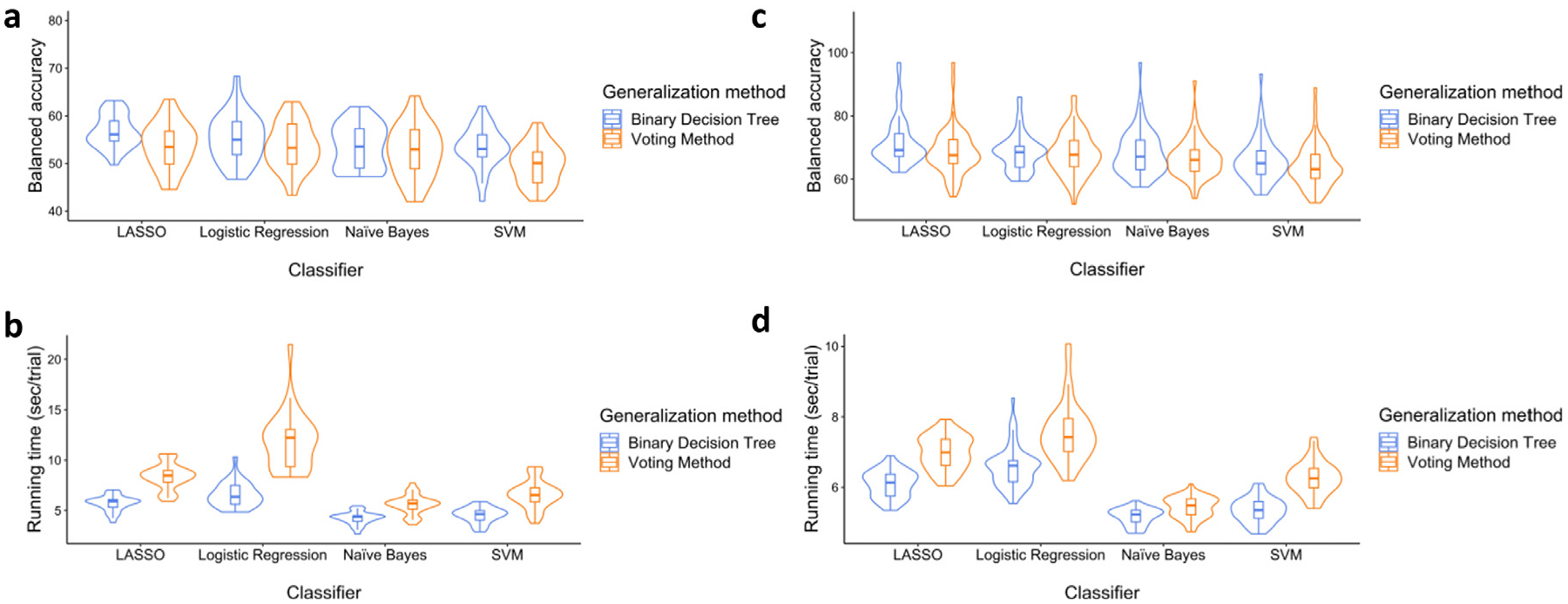
Comparisons of balanced accuracy and running time for different feature selection methods and classifiers for multiclass classification problems. **A)** balanced accuracy for context memory; and **B**) running time (in seconds per trial) for context memory; **C**) balanced accuracy for context perception; **D**) running time (in seconds per trial) for context perception. The violin plots indicate the distribution of data scores. The box plots are shown inside the violin plots. The average chance levels—the average of the 95^th^ percentile of the balanced accuracy values in the null distribution—across participants were in the range of 25.2 to 27.8% for the 4-class analyses and in the range of 33.6 to 36.4% for the 3-class analyses. It should be noted that classification performances were significantly above the empirical chance levels across the participants for these analyses.

In this problem, we were interested in classifying all four different types of color context memory states including correct with high confidence, correct with low confidence, incorrect with low confidence, and incorrect with high confidence. One important thing to keep in mind is that there were only 29 participants who had more than 20 trials for each of these four classes and we performed the classification only for these participants. The summary of the results can be found in Figures 6a and 6b.

To statistically compare generalization methods and classifiers, we ran a Generalization (voting, binary decision tree) × Classifier (LASSO, logistic regression, naïve Bayes, and SVM) ANCOVA for both balanced accuracy and running time with age as a covariate. For balanced accuracy, significant main effects of Generalization [*F*(1, 223) = 14.57, *p* < 0.001, $\eta_{p}^{2}=0.061$] and Classifier [*F*(3, 223) = 7.47, *p* < 0.001, $\eta_{p}^{2}=0.091$] were observed after controlling for Age, which was a significant predictor of accuracy and it was associated with lower accuracy [*F*(1, 223) = 8.05, *p* < 0.001, $\eta_{p}^{2}=0.035$]. The interaction was not significant [*F*(3, 223) = 0.80, *p* = 0.480, $\eta_{p}^{2}=0.011$]. As can be seen in Figure 6a, generalization using the binary decision tree outperformed the voting method. Moreover, follow-up t-tests showed that LASSO outperformed the other classifiers [all *t*s > 3.21, *p*s < 0.002] except logistic regression [*t*(28) = 1.20, *p* = 0.119].

For running time, main effects of generalization [*F*(1, 223) = 244.87, *p* < 0.001, $\eta_{p}^{2}=0.523$] and classifier [*F*(3, 223) = 110.01, *p* < 0.001, $\eta_{p}^{2}=0.597$] and the interaction [*F*(3, 223) = 22.33, *p* < 0.001, $\eta_{p}^{2}=0.231$] were all significant after controlling for Age, which was not [*F*(1, 223) = 2.85, *p* = 0.093, $\eta_{p}^{2}=0.013$],. Follow-up t-tests indicated that the binary decision tree generalization method was faster than the voting method for all classifiers [all *t*s > 11.04, *p*s < 0.001]. As can be seen in Figure 6b, naïve Bayes was faster than the other classifiers [all *t*s > 5.11, *p*s < 0.001].

Lastly, classifier performance was not a significant predictor of context d-prime [*ρ*(26) = −0.172, *p* = 0.382]. For this analysis, we computed the partial Pearson correlation between context d-prime and the highest-performing classification method (using LASSO when the binary decision tree generalization method was used) while controlling for age.

#### 3.2.2. Three-Class Classification of Context Perception

In this problem, we were interested in classifying the trials based on the color context that the participant perceived during encoding (i.e., red vs. green vs. brown). We used only the trials where the object was correctly identified as old, and the color context was correctly identified. The results are shown in Figures 6c and 6d^2^.

In order to statistically compare generalization methods and classifiers, we ran a Generalization (voting, binary decision tree) × Classifier (LASSO, logistic regression, naïve Bayes, and SVM) ANCOVA for balanced accuracy, controlling for Age. For balanced accuracy, significant main effects of Generalization [*F*(1, 471) = 8.96, *p* = 0.003, $\eta_{p}^{2}=0.019$] and Classifier [*F*(3, 471) = 12.13, *p* < 0.001, $\eta_{p}^{2}=0.072$] were observed after controlling for Age, which was a significant negative predictor of accuracy [*F*(1, 471) = 11.08, *p* < 0.001, $\eta_{p}^{2}=0.023$]. The interaction was not significant [*F*(3, 471) = 0.75, *p* = 0.524, $\eta_{p}^{2}=0.005$]. As can be seen in Figure 6c, generalization using the binary decision tree outperformed the voting method. Moreover, follow-up t-tests showed that LASSO outperformed the other classifiers [all *t*s > 5.08, *p*s < 0.001].

For running time, main effects of generalization [*F*(1, 471) = 311.22, *p* < 0.001, $\eta_{p}^{2}=0.398$], classifier [*F*(3, 471) = 320.30, *p* < 0.001, $\eta_{p}^{2}=0.671$], as well as the interaction [*F*(3, 471) = 15.43, *p* < 0.001, $\eta_{p}^{2}=0.089$] were all significant after controlling for Age which was associated with higher running time [*F*(1, 471) = 4.88, *p* = 0.028, $\eta_{p}^{2}=0.010$], Follow-up t-tests to elucidate the source of interaction showed that the binary decision tree generalization method was faster than the voting method for all classifiers [all *t*s > 9.82, *p*s < 0.001]. As can be seen in Figure 6d, naïve Bayes was faster than the other classifiers for the binary decision tree [all *t*s > 6.32, *p*s < 0.029] and voting method [all *t*s > 19.39, *p*s < 0.001].

### 3.3. Testing the generalizability of the methodology

The above analyses showed that the optimal classification procedure, considering both performance and running time, was obtained by first extracting a diverse feature space, filtering a subset of top performing features, and passing them to the wrapper for feature selection. While the classifiers performed similarly, LASSO tended to outperform the others by a small margin—by at least 5.8%-points compared to the other classifiers—, and naïve Bayes tended to be fastest—other classifiers had at least 7.1% increase (0.17 sec/trial) in processing time. However, it is essential to verify that this approach works well for other classification problems and datasets in order to establish its generalizability. To this end, we performed similar analyses for new datasets from different cognitive domains: an EEG working memory dataset collected from patients with frontal lobe injury and age-matched controls; a motor imagery EEG dataset, and an MEG working memory dataset (see Code and data accessibility in Materials and Methods). We believe that diversity in terms of the signal type, cognitive domain, data dimensionality, and the healthiness of the participants’ brains could be substantially supportive of the generalizability of our findings.

**Figure 7.**
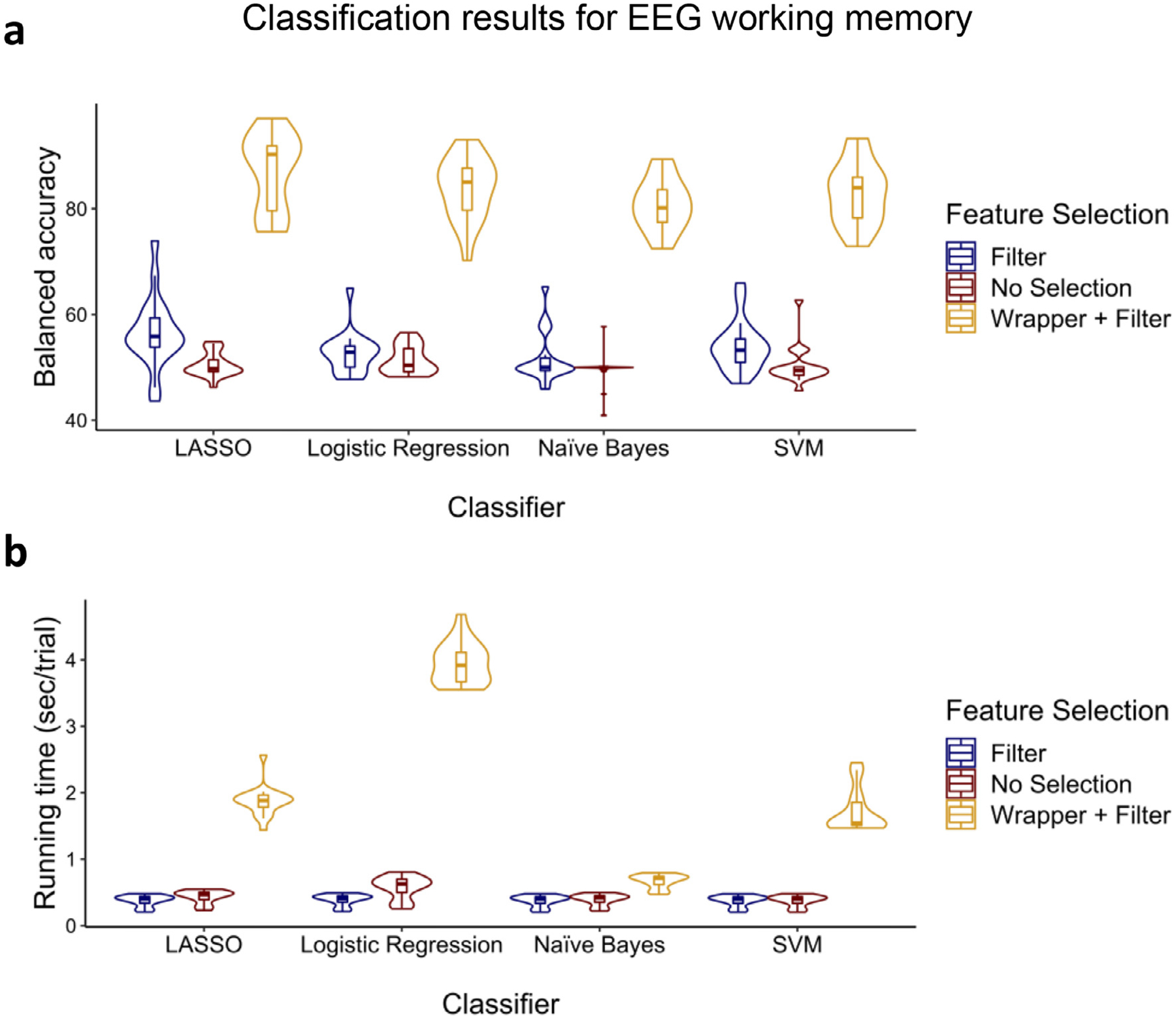
Comparisons of balanced accuracy and running time for different feature selection methods and classifiers for EEG working memory: **A)** balanced accuracy; and **B)** running time (in seconds per trial). The violin plots indicate the distribution of data scores. The box plots are shown inside the violin plots. The average chance levels—the average of the 95^th^ percentile of the balanced accuracy values in the null distribution—across participants were in the range of 50.4 to 53.3% for all analyses. It should be noted that classification performances were significantly above the empirical chance levels across the participants for these analyses.

In the EEG working memory study, participants were asked to encode two colored shapes and following a short delay, asked to make shape identity, spatial location, and temporal order discriminations about shape probes (Johnson et al., 2017). EEG data were collected in this study across 64 electrodes from 14 patients with focal prefrontal cortex damage and 20 aged-matched controls, ranging in age from 30–62. Although there were additional subjects in this study, we only used the data from those (i.e., 12 patients and seven controls) with at least 20 trials in each condition/class. We were interested in classifying the trials in which the participant had responded correctly versus incorrectly across identity, spatial location, or temporal order discriminations. The summary of the classification accuracy and running time results can be found in Figure 7.

To statistically compare feature selection methods and classifiers, we ran a Feature selection (filter, filter + wrapper, no selection) × Classifier (LASSO, logistic regression, naïve Bayes, and SVM) ANOVA for both balanced accuracy and running time. For balanced accuracy, significant main effects of feature selection [*F*(2, 216) = 1071.70, *p* < 0.001, $\eta_{p}^{2}=0.908$] and classifier [*F*(3, 216) = 6.43, *p* < 0.001, $\eta_{p}^{2}=0.082$] were observed. However, the interaction [*F*(6, 216) = 1.47, *p* = 0.191, $\eta_{p}^{2}=0.039$] was not significant. Follow-up t-tests showed that the filter + wrapper selection method outperformed the other two selection methods [all *t*s > 18.15, *p*s < 0.001] while filter alone outperformed no selection [*t*(18) = 30.89, *p*s = 0.003]. Moreover, as can be seen in Figure 7a, LASSO outperformed the other classifiers [all *t*s > 3.16, *p*s < 0.003].

For running time, main effects of feature selection [*F*(2, 216) = 84.93, *p* < 0.001, $\eta_{p}^{2}=0.440$], classifier [*F*(3, 216) = 17.69, *p* < 0.001, $\eta_{p}^{2}=0.197$], and the interaction [*F*(6, 216) = 14.70, *p* < 0.001, $\eta_{p}^{2}=0.290$] were all significant. Follow-up t-tests confirmed that the filter selection method was faster than the other two methods [all *t*s > 3.93, *p*s < 0.001] while no selection was faster than the filter + wrapper [all *t*s > 3.92, *p*s < 0.001] for all classifiers. As can be seen in Figure 7b, naïve Bayes was faster than the others for the filter + wrapper method [all *t*s > 3.06, *p*s < 0.004]. For the filter method, SVM was faster than the others [all *t*s > 8.49, *p*s < 0.001] except naïve Bayes [*t*(18) = 1.55, *p* = 0.070]. Lastly, for no selection, SVM was faster than the others [all *t*s > 9.19, *p*s < 0.001].

**Figure 8.**
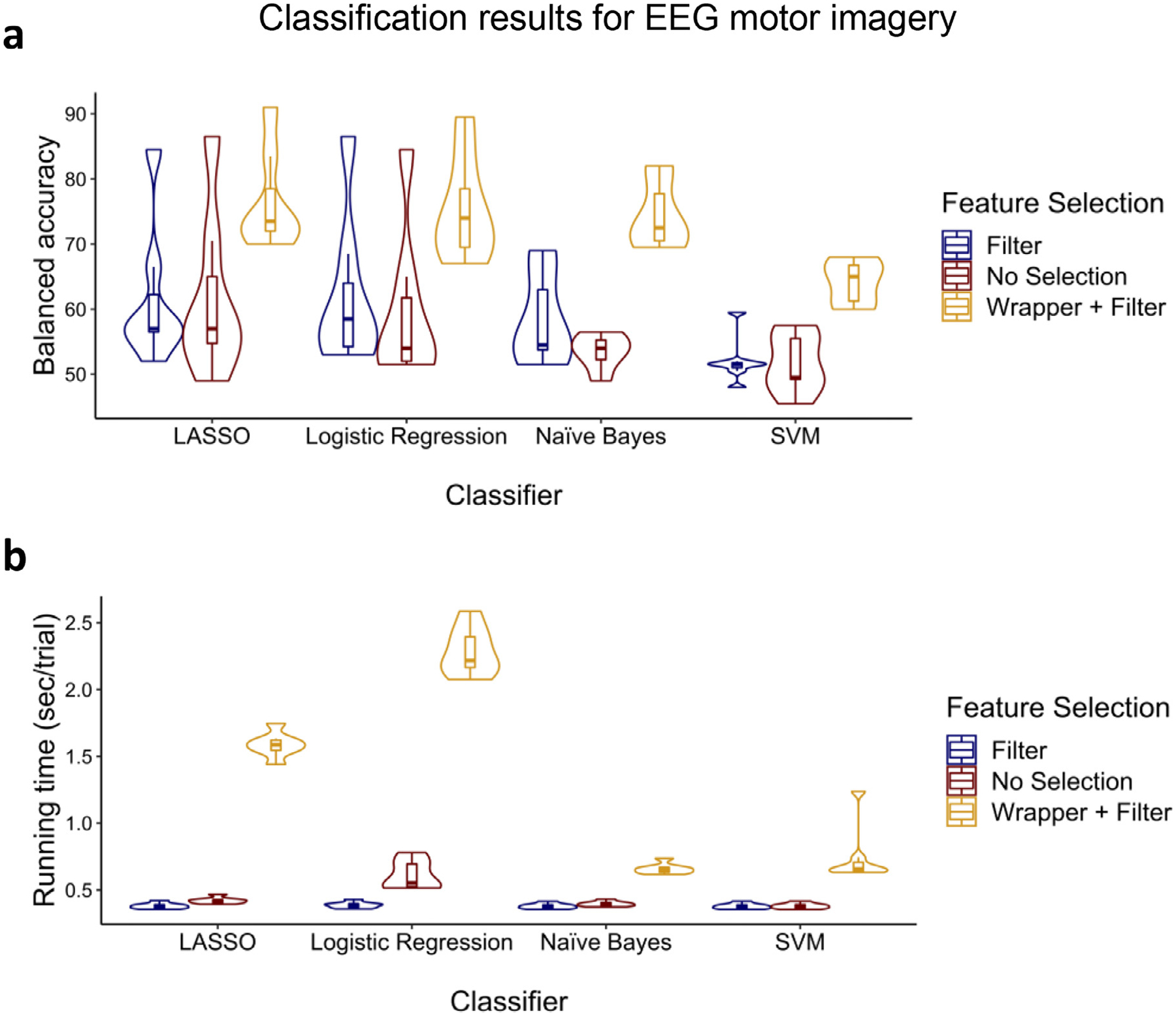
Comparisons of balanced accuracy and running time for different feature selection methods and classifiers for EEG motor imagery: **A)** balanced accuracy; and **B)** running time (in seconds per trial). The violin plots indicate the distribution of data scores. The box plots are shown inside the violin plots. The average chance levels—the average of the 95^th^ percentile of the balanced accuracy values in the null distribution—across participants were in the range of 50.6 to 53.2% for all analyses. It should be noted that classification performances were significantly above the empirical chance levels across the participants for these analyses.

In the second analysis, we performed classification on data collected during performance of a motor imagery task in which participants had to imagine moving their left hand or their right hand (Blankertz, Dornhege, Krauledat, Müller, & Curio, 2007). EEG data were collected in this study across 59 electrodes from seven healthy adults aged 26 to 46. We classified right hand movement versus left hand movement trials. We repeated the same procedure that we did for the previous analysis and the summary of the results can be found in Figure 8.

A Feature selection (filter, filter + wrapper, no selection) × Classifier (LASSO, logistic regression, naïve Bayes, and SVM) ANOVA for balanced accuracy showed significant main effects of feature selection [*F*(2, 72) = 30.85, *p* < 0.001, $\eta_{p}^{2}=0.462$] and classifier [*F*(3, 72) = 7.08, *p* < 0.001, $\eta_{p}^{2}=0.228$] but no significant interaction [*F*(6, 72) = 0.36, *p* = 0.904, $\eta_{p}^{2}=0.029$]. Follow-up t-tests showed that the filter + wrapper selection method outperformed the other two methods [all *t*s > 12.15, *p*s < 0.001] while there was no difference between the filter alone and no selection [*t*(6) = 1.57, *p*s = 0.084]. Moreover, as can be seen in Figure 8a, LASSO outperformed SVM [*t*(6) = 3.28, *p* = 0.008] but did not outperform other two classifiers [all *t*s < 1.56, *p*s > 0.085].

For running time, main effects of feature selection [*F*(2, 72) = 31.07, *p* = 0.001, $\eta_{p}^{2}=0.463$] and classifier [*F*(3, 72) = 11.65, *p* < 0.001, $\eta_{p}^{2}=0.327$] and the interaction [*F*(6, 72) = 9.35, *p* < 0.001, $\eta_{p}^{2}=0.438$] were all significant. Follow-up t-tests confirmed that the filter selection method was faster than the other two methods [all *t*s > 2.90, *p*s < 0.014] while no selection was faster than the filter + wrapper [all *t*s > 3.58, *p*s < 0.006] for all classifiers. As can be seen in Figure 8b, naïve Bayes was faster than the others for the filter + wrapper method [all *t*s > 3.46, *p*s < 0.007] except SVM [*t*(6) = 1.03, *p* = 0.172]. For the filter method, SVM was faster than the others [all *t*s > 4.04, *p*s < 0.001] except naïve Bayes [*t*(6) = 1.34, *p* = 0.114]. Lastly, for no selection, SVM was faster than the others [all *t*s > 6.33, *p*s < 0.001].

**Figure 9.**
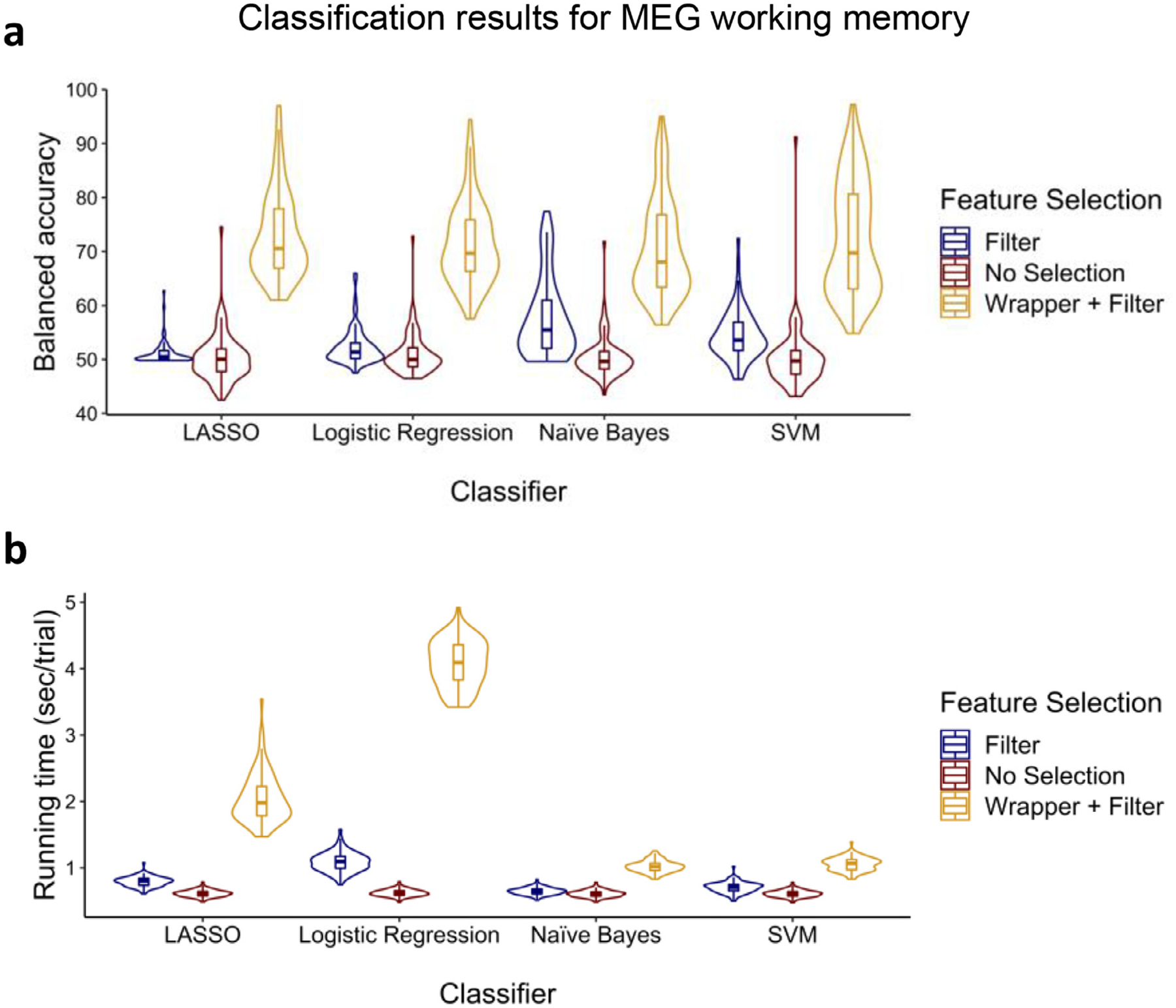
Comparisons of balanced accuracy and running time for different feature selection methods and classifiers for MEG working memory: **A)** balanced accuracy; and **B)** running time (in seconds per trial. The violin plots indicate the distribution of data scores. The box plots are shown inside the violin plots. The average chance levels—the average of the 95^th^ percentile of the balanced accuracy values in the null distribution—across participants were in the range of 50.2 to 53.6% for all analyses. It should be noted that classification performances were significantly above the empirical chance levels across the participants for these analyses.

In the third analysis, we used the Human Connectome Project dataset (Larson-Prior et al., 2013). We performed classification on MEG signals recorded during a working memory task with alternating 0-back and 2-back conditions in which participants were presented with pictures of tools or faces. MEG data were collected in this study across 248 electrodes from 83 healthy adults aged 22 to 35. We classified correct responses versus incorrect responses. We repeated the same procedure that we did for the previous analyses and the summary of the results can be found in Figure 9.

A Feature selection (filter, filter + wrapper, no selection) × Classifier (LASSO, logistic regression, naïve Bayes, and SVM) ANOVA for balanced accuracy showed significant main effects of feature selection [*F*(2, 984) = 1099.58, *p* < 0.001, $\eta_{p}^{2}=0.691$] and classifier [*F*(3, 984) = 2.82, *p* = 0.038, $\eta_{p}^{2}=0.009$] and the interaction [*F*(6, 984) = 8.91, *p* < 0.001, $\eta_{p}^{2}=0.052$]. Follow-up t-tests showed that the filter + wrapper selection method outperformed the other two methods [all *t*s > 12.54, *p*s < 0.001], and filter alone outperformed no selection [all *t*s > 3.08, *p*s < 0.002] for all classifiers except LASSO [*t*(82) = 1.63, *p* = 0.053]. Moreover, as can be seen in Figure 9a, LASSO outperformed the other classifiers for the filter + wrapper method [all *t*s > 4.43, *p*s < 0.001] except SVM [*t*(82) = 1.00, *p* = 0.160]. For the filter method, naïve Bayes outperformed others [all *t*s > 5.05, *p*s < 0.001]. Lastly, for no selection, logistic regression outperformed others [all *t*s > 2.52, *p*s < 0.007].

For running time, main effects of feature selection [*F*(2, 984) = 1026.26, *p* < 0.001, $\eta_{p}^{2}=0.676$] and classifier [*F*(3, 984) = 375.71, *p* < 0.001, $\eta_{p}^{2}=0.534$] and the interaction [*F*(6, 984) = 254.94, *p* < 0.001, $\eta_{p}^{2}=0.609$] were all significant. Follow-up t-tests confirmed that the filter selection method was faster than the other two methods [all *t*s > 17.86, *p*s < 0.001] while no selection was faster than the filter + wrapper [all *t*s > 21.57, *p*s < 0.001] for all classifiers. As can be seen in Figure 9b, naïve Bayes was faster than the others for the filter + wrapper method and the filter method alone [all *t*s > 6.73, *p*s < 0.001]. Lastly, for no selection, naïve Bayes was faster than the others [all *t*s > 5.38, *p*s < 0.001] except SVM [*t*(82) = 0.84, *p* = 0.203].

It is important to note that performance for the MEG dataset was generally lower than was performance for the EEG working memory dataset, although both datasets were collected from working memory tasks. Some of the potential reasons include the potential differences in the sensitivity of MEG and EEG to certain neural signals, or the differences in MEG and EEG measurement and/or preprocessing, as well as the differences in the paradigms used in the two datasets. However, a potential mathematical reason for this difference is related to the number of features the wrapper has considered compared to the overall feature space. Specifically, for the sake of running time, the wrapper considered only 100 features for finding the optimal feature set for each analysis. While 100 features are about 6% of the total extracted features for the EEG working memory dataset, they are only about 2% of the total extracted features for the MEG working memory dataset due to the MEG recording having more channels. As a result, this could potentially impact the classification performance for the MEG dataset as it could not explore more features because of running time (See supplementary material for “Impact of the number of features”).

### 3.4. Systematic comparison of feature types

For each problem, we investigated how frequently each feature type was selected by feature selection algorithms across all subjects. Supplementary Tables 1–4 provide a summary of how frequently different feature types, time windows, frequency bands, and electrode regions were selected in the feature selection process across subjects and different analyses. These tables indicate that CSP features were selected more than other types of features while features from delta frequency band were selected more often than other frequency bands. It is important to note that just because a particular type of feature has been selected more frequently than another, it does not necessarily indicate that it yields better classification performance. For example, features that are efficient for classification but fail to be in the top X% will not pass the Fisher criterion for being passed to the classifier. To address this issue, each classifier was provided with only a specific type of feature (e.g., only entropy features or only CSP-based features, etc.) and classification performances were compared. The result of these comparisons is shown in Table 1. These results are obtained by using the combination of filter and wrapper methods to select the effective features and then training naïve Bayes classifiers on all subjects in all analyses. We repeated the process for other methods for only 10 subjects (it was not practical to repeat them for all subjects because of high computational complexity) and the patterns were similar to naïve Bayes, hence we have not shown them in the table. The Feature type (CSP-based, mean, variance, entropy, AR coefficients, correlations, phase, phase synchrony, and multiple types together) ANOVAs indicated that there is a significant difference between the performances of different feature types [item memory : *F*(8, 531) = 30.21, *p* < 0.001, $\eta_{p}^{2}=0.231$, context decoding: *F*(8, 252) = 18.47, *p* < 0.001, $\eta_{p}^{2}=0.218$, context memory: *F*(8, 252) = 13.64, *p* < 0.001, $\eta_{p}^{2}=0.302$, EEG working memory: *F*(8, 162) = 3.20, *p* = 0.002, $\eta_{p}^{2}=0.136$, motor imagery: *F*(8, 54) = 21.21, *p* < 0.001, $\eta_{p}^{2}=0.759$, MEG working memory: *F*(8, 738) = 19.06, *p* < 0.001, $\eta_{p}^{2}=0.171$]. Moreover, extracting multiple types of features outperformed extracting only an individual type of feature for all feature types in all analyses [item memory : all *t*s > 3.86, *p*s < 0.004)] context decoding: all *t*s > 3.10, *p*s < 0.002), context memory: all *t*s > 3.74, *p*s < 0.002), EEG working memory: all *t*s > 2.11, *p*s < 0.025), motor imagery: all *t*s > 2.90, *p*s < 0.014), MEG working memory: all *t*s > 6.34, *p*s < 0.001)].

## 4. Discussion

In everyday life, even a person with no clinically significant memory impairment shows episodic memory failures such as forgetting where they parked their car. Using fMRI and EEG, cognitive neuroscientists have been examining the neural foundations of these kinds of memory failures, and successes, with various approaches. While it is common to use ERPs or average BOLD signals to discriminate neural activity associated with successful versus unsuccessful memory performance, averaging approaches do not allow us to explore single events. Classification of brain states associated with single events using real-time signals recorded from the scalp offers the potential for development of real-time interventions to support everyday learning. Although some studies have performed single trial classification of different memory states, performance has arguably been insufficient for developing an effective intervention system. To address this problem, in this study, for the first time, we systematically compared different methods for each step of classification for the same dataset collected from an adult lifespan sample to examine which combination of methods offers the best performance. This study has several novel aspects. Specifically, we inspected different methods at all steps of classification for various episodic memory and visual perception cognitive problems with different numbers of classes and levels of imbalance on an adult lifespan dataset. Moreover, we investigated the generalizability of our findings by applying our methods on three other datasets from working memory and motor imagery domains. Our results suggest that no particular feature type outperforms others consistently, and it is important to extract multiple types of features to have an optimal performance as the ANOVAs in the beginning of the Results section showed. Moreover, the combination of the wrapper and filter methods outperformed the filter method, and both outperformed no feature selection method. Additionally, although different classifiers performed at a similar level, LASSO was consistently the highest performing while naïve Bayes was the fastest classifier. Lastly, for multiclass classification, the best strategy to generalize the binary classification is to use binary decision trees. We elaborate on these results below.

### 4.1. Recommendations for Future Studies

Researchers who want to optimize their classification performance can leverage the current results to make informed decisions regarding the specific feature extraction, feature selection, and classification methods to select for their problem of interest. Here, we have provided the answers to a list of likely questions that a researcher will need to answer before performing classification analyses. It is worth mentioning that while we make these recommendations for EEG/MEG datasets, some of the principles, particularly the ones derived mathematically and independent of the type of the investigated time series, may apply to other kinds of data, including fMRI. Specifically, the recommendations regarding feature extraction, feature selection, imbalance handling, and number of features to search through and select may apply to fMRI as well.

#### 4.1.1. What type(s) of features should I extract?

Regarding the features that one should extract, it is highly recommended to extract as many types of feature as possible (al-Qerem, Kharbat, Nashwan, Ashraf, & blaou, 2020). Importantly, we cannot recommend a particular type of feature for the researcher to extract as no feature type stood out in our systematic analyses for each cognitive problem. However, we did find that extracting many types of features outperformed extracting only one feature type. Each cognitive problem likely reflects a particular combination of neural activities and different feature types might hold different levels of importance for that particular problem. While extracting many types of features (i.e., entropy, phase synchronization, correlation, CSP-based features, etc.) outperformed extracting any specific feature (see Table 1), one might wonder why one would use only a specific type of feature or a specific set of features in their study. A researcher might be interested in features that are an inherent property of the brain, so the findings can be more directly related to this property. For example, Höhne et al., 2016 used both oscillatory EEG phase and power information and compared their associated performances; researchers found that classification using phase features outperformed that using power features. These results allowed the researchers to endorse the functional relevance of phase for long-term memory operations and recommended that phase information might be utilized for memory enhancement applications that use deep brain stimulation. It is also possible that some features might be particularly diagnostic for classifying disease states. For example, one might want to use classification analyses to identify Alzheimer’s pathology or to assess progression of the disease. EEG, being non-invasive, inexpensive, and widely available in clinical settings, holds potential for diagnosis of Alzheimer’s disease (Vecchio et al., 2013). Some evidence shows that entropy is altered by Alzheimer’s disease and is able to distinguish patients from healthy controls (Nobukawa et al., 2020). Of course, it remains possible that extraction of additional features could produce better classification performance. Thus, the choice of features to extract may depend upon the purpose of the researcher’s classification problem but as we have shown here, multiple feature extraction may prove superior for classifying several types of cognitive problems.

#### 4.1.2. How should I select the features that I want to use to train the classifier?

While it is crucial to get as much information as possible from each trial by extracting several types of features across time, frequency bands, and electrodes, not all the extracted features will necessarily be useful for classification. There are two commonly used techniques for finding the effective features for classification—namely, filtering and wrapper methods while an alternative and control method would be to not perform feature selection at all. Filtering methods are fast since they involve a non-iterative computation on the dataset which execute faster than training a classifier. Moreover, since the filter methods evaluate the intrinsic characteristics of the features, rather than their interaction with a specific classifier, they will produce performance that is more similar across classifiers than the wrapper method (as can be seen in Figures 5 and 7–9). By contrast, wrapper methods generally achieve higher performance than filter methods, but performance is dependent upon interactions between the classifier and the subset of features. Moreover, the fact that the wrapper method evaluates several different sets of features by directly training the classifier using those features explains its slower execution (Mitchell, 1997). If the number of extracted features is high, it will be impractical to pass all of the features to the wrapper; it would be wise to apply wrapper methods on a smaller set of features by first using filter methods to score individual features, then picking those with the highest scores to pass to the wrapper (see Supplementary Figure 1). With these issues in mind, for the classification problems for which accuracy is the most important factor, wrapper methods such as sequential forward selection should be used to select the most effective features (Dias, Kamrunnahar, Mendes, Schiff, & Correia, 2010; Kirar & Agrawal, 2018; Zhang, Gan, & Wang, 2015). In the current analyses, on average, selecting the effective features using the combination of filter and wrapper methods resulted in 36.1%-points balanced accuracy improvement with a 173.2% increase (1.55 sec/trial) in processing time compared to the filter method alone. Moreover, the filter method alone resulted in 5.8%-points balanced accuracy improvement with a 0.4% decrease (2 ms/trial) in processing time compared to no feature selection at all (see Figures 5 and 7–9).

#### 4.1.3. Which classifier should I use?

An important point of emphasis is that all the classifiers commonly used in the literature perform relatively well; otherwise, they would not be used in those studies. As mentioned previously, each of these classifiers uses a particular and unique strategy to distinguish the data points and has its strengths and weaknesses. Specifically, all of the classifiers performed similarly, although there were some minor differences in their achieved accuracy. It is fair to say that the choice of classifier may not be as determinant as other factors in terms of the performance. In this study, in terms of balanced accuracy, we found that on average across the classification problems, LASSO outperformed logistic regression by 4.1%-points, logistic regression outperformed naïve Bayes by 2.2%-points, and naïve Bayes outperformed SVM by 0.2%-points. One advantage of LASSO is the way it optimizes the problem by potentially removing some of the selected features during training and testing the model’s performance (i.e., mean squared error) of different combinations and sizes of feature sets. While this adds to the running time, it can improve classification performance relative to other classifiers by potentially having a simpler and more efficient model.

However, if a researcher is highly concerned about the running time, as can be seen in Figures 5–9, naïve Bayes would be the best choice as it is remarkably faster than the other classifiers, and it performs closely to the highest performing classifiers (i.e., LASSO and then logistic regression). In this study, we found that on average across the cognitive classification problems, SVM had 14.8% increase (0.29 sec/trial) in processing time compared to naïve Bayes, LASSO had 30.7% increase (0.69 sec/trial) in processing time compared to SVM, and logistic regression had 45.7% increase (1.34 sec/trial) in processing time compared to LASSO. The parameters of the naïve Bayes model including a priori and conditional probabilities are learned using a deterministic set of steps. These steps involve only counting and dividing which are trivial operations. Moreover, as we mentioned above, naïve Bayes does not perform an optimization of a cost equation involved in training the model and does not solve any matrix equations which are procedures that can be computationally costly (Fukunaga, 1990), as can be seen for LASSO and logistic regression. On the contrary, logistic regression requires more data to reach stability, and for some participants with lower numbers of trials, execution of its training is slow (as can be seen in Figures 5–9).

#### 4.1.4. What if the number of trials is not the same across different classes?

It is important to handle the imbalance issue to prevent the classifier from having any skewed tendency to label the test data as the overrepresented class. While there are different strategies such as under-sampling (e.g., bootstrap aggregation or “bagging”) and over-sampling (e.g., SMOTE) to handle the imbalance issue, we recommend using the SMOTE technique to train the classifier with an equal number of trials from each class. As can be seen in Supplementary Figure 2, we found that on average across different classifiers, SMOTE outperformed the approach in which we did not handle the imbalance issue by 10.5%-points in terms of balanced accuracy. Interestingly, not handling the imbalance outperformed the bagging method by 7.7%-point. In terms of running time, the SMOTE method had 26.7% increase (0.42 sec/trial), and the bagging method had 531.9% increase (9.38 sec/trial) in processing time compared to not handling the imbalance. Although SMOTE adds a computation cost to the analyses, the improvement that it will have in terms of performance and removing any imbalance during the training stage of the classifier is arguably worth the extra computation cost.

#### 4.1.5. How many features should I search through and how many features should I select?

Two important parameters that one needs to determine are the number of features to include in the feature space for the wrapper after filtering and the number of top-performing features that one should select to properly train the classifier. These choices likely depend on the problem, how many trials are available, the level of noise in the dataset, and one’s tolerance of the running/computing time. It is recommended to plot a figure like Supplementary Figure 1 to understand how much performance would change as a function of these two parameters. As can be seen in the figure, performance increases as more features are provided to the wrapper and then plateaus. On the other hand, performance increases when increasing the number of selected features, plateaus, and then begins to decrease due to overfitting. Moreover, the running time increases linearly when providing more features to the wrapper or increasing the number of selected features. The cutoff point will change based on the problem and researcher’s preference and represents a potential trade-off between performance and running time. Specifically, there is not a unique cutoff point on which all researchers would agree in determining these two parameters. For example, if running time is not a concern for the researcher, they might decide to pass more features to the wrapper to reach even a slightly higher performance compared to the point in which the plot is starting to plateau. As the specific number of features and running time will likely differ between studies, we recommend researchers examine figures like Supplementary Figure 1 to make informed determinations regarding feature number.

#### 4.1.6. What if I am interested in classifying more than two cognitive conditions?

To classify more than two classes, the problem must be broken into multiple binary classification problems. While there are several approaches to doing so, the efficient way—in terms of both running time and performance—is to use binary decision trees to generalize the binary problem into a multiclass one. While binary decision trees have been frequently used for this purpose (Fei & Liu, 2006; Freeman, Kuli, & Basir, 2013; Mao, Zhou, Pi, Sun, & Wong, 2005), in this study, we also found that they outperform the voting method which is also commonly used in the literature (Khan, Bhatti, Khan, & Iqbal, 2019; Krishna, Pasha, & Savithri, 2016; Mahmoudi & Shamsi, 2018; Venate & Sunny, 2016). Specifically, on average, generalizing a binary classification problem into a multiclass problem using binary decision trees resulted in 4.5%-point improvement in balanced accuracy and 32.5% decrease (1.04 sec/trial) in processing time compared to generalizing using the voting method as can be seen in Figure 6. For binary decision trees, the executions are faster as fewer classification analyses are conducted, and when the results of the binary problems are combined to produce the final label, binary decision trees will have better performance as the errors of the binary problems will be accumulated to some degree to generate the final labels (Fei & Liu, 2006).

### 4.2. Impact of aging on classification performance

It is important to note that chronological age did not influence classification performance for the item memory (i.e., item hit vs. miss) classifier, consistent with the behavioral results showing relatively spared item memory discriminability across age [the relationship between age and item memory: *ρ*(58) = −0.144, *p* = 0.273]. By contrast, age had a significant negative effect on 4-class context memory decoding, which again fit behavioral performance for context memory d-prime [the relationship between age and context memory: *ρ*(58) = −0.412, *p* = 0.001]. Finally, age had a significant negative effect on 3-class context perception classifier. While we did not explicitly measure visual perception performance in our memory study, many previous studies have shown perceptual processing deficits in normal aging (Monge & Madden, 2016; Roberts & Allen, 2016). Importantly, despite age-related reductions in the level classification accuracy, the patterns of results for feature extraction, feature selection, and classifier selection were age-invariant. Thus, the recommendations we offer here apply to participants across the adult lifespan.

### 4.3. Limitations of this study

The results of this study should be interpreted in the context of some limitations. First, despite the richness of the selected dataset, which allowed us to perform multiple classification analyses, it lacks simplicity that could have allowed for higher classification performance. That is, for each trial, participants were asked to pay attention to multiple pieces of information including objects, scene and color contexts, and their relationships. Consequently, brain activity likely reflects multiple, complex cognitive operations. However, by validating our methodological approach on two orthogonal datasets including one collected from a very simple motor imagery design, we have shown that our approach works well regardless of the complexity of the task or cognitive domain.

Although we assessed feature types, feature selection methods, and classifiers that have commonly been used in cognitive neuroscience studies, there are many other choices, and it would be worth investigating them in future studies. Lastly, it is important to note that performances in this study are not necessarily the best that could be achieved. That is, we determined the parameters such as the number of features passed to the wrapper and the number of features the wrapper selects according to the trade-off between running time and performance. Although this is the approach most researchers would likely take, by sacrificing the running time or using super computers, it could be possible to reach better performance (as can be seen in Supplementary Figure 1).

### 4.4. Future directions

One of the motivations for single-trial classification is to provide real-time and real-world interventions using brain-computer interfaces. While BCIs have been implemented successfully in applications such as smart wheelchairs and robotic exoskeletons, such systems have not been practically implemented in the memory domain, yet there have been some related attempts (Burke, Merkow, Jacobs, Kahana, & Zaghloul, 2015; DeBettencourt, Cohen, Lee, Norman, & Turk-Browne, 2015; Ezzyat et al., 2018). In this regard, this study provides some recommendations to improve classification performance, which is important as even minor improvements in a classifier’s performance can potentially result in practically and clinically meaningful developments in BCI device performance.

While classification performance in this study outperformed any previous non-invasive EEG studies of memory by between 5–12% (Astrand, 2018; Noh et al., 2014, 2018), addressing the aforementioned limitations could potentially increase performance even further. Ideally, the desired performance of a BCI system is 100%, which is almost certainly out of reach, as scalp EEG signals are noisy, and distinguishing some cognitive states might be difficult no matter how efficient the chosen methodology. As a result, sufficient performance is debatable, and it largely depends upon the specific problem, how costly a wrong prediction will be, and the preferences of the researchers and the individuals who intend to use the potential device. For example, for the hypothetical learning facilitator, even 80% accuracy may be valuable.

## 5. Conclusion

Using EEG recorded during performance of an episodic memory task in an adult lifespan sample, we systematically compared different methods of feature extraction, feature selection, and classifier in the same study to examine which methods work the best for various binary and multiclass classification problems. We found that no feature type outperforms others on a consistent basis, and it is crucial to extract multiple types of features to reach an optimal performance. The combination of filtering and sequential forward selection was the optimal method to select the effective features. Furthermore, although the classifiers performed close to each other, LASSO was generally the highest-performing option while naïve Bayes was faster than the other common options. Moreover, we tested these methods on three other datasets, and they outperformed alternative methods, supporting their generalizability. These findings are of remarkable value for understanding how the chosen method at each step of classification influences how efficiently different cognitive states can be discriminated and for selecting the most appropriate classification procedure in relevant investigations. In conclusion, we believe that our recommendations could provide a fruitful insight for cognitive researchers interested in performing single-trial classification and can be translated into the clinical realm for real-time detection of the cognitive states and providing the suitable interventions when necessary.

## Supplementary Material

1

## Figures and Tables

**Table 1:** Classification performance for each feature type across all participants for each cognitive problem. For each column, only the associated type of feature was used for classification. The last column represents including multiple types (all of the 8 feature types mentioned in this table) of features together.

	CSP	Mean	Variance	Entropy	AR	Correlation	Phase	Phase synchrony	Multiple types
Item memory	70.7%	70.1%	70.2%	70.5%	63.0%	68.2%	70.4%	69.3%	72.4%
3-class context perception	65.9%	65.3%	65.1%	66.3%	55.8%	62.8%	65.5%	62.4%	68.2%
4-class context memory decoding	52.0%	51.9%	51.3%	51.9%	44.6%	50.1%	51.7%	50.6%	53.5%
EEG working memory	78.2%	77.3%	76.9%	73.7%	73.8%	73.5%	75.2%	74.4%	80.7%
Motor imagery	72.1%	65.5%	63.6%	58.4%	59.1%	58.8%	57.5%	58.3%	74.4%
MEG working memory	67.8%	67.9%	67.6%	67.5%	67.4%	67.3%	67.1%	67.4%	69.6%

## Footnotes

1 The same analysis for scene perception revealed similar results.

2 Similar results were obtained from the classification analyses for scenes (i.e., studio apartment, cityscape, or island).
