# Supplementary material for: Evaluation of classification approaches for distinguishing brain states predictive of episodic memory performance from electroencephalography
Source: Neuroimage. Author manuscript; Available in PMC 2022 Feb 15. (PMC8824531; doi:10.1016/j.neuroimage.2021.118851)
Supplement: 1 [file NIHMS1774998-supplement-1.docx]

*Measures Used to Assess a Classifier’s Performance*

To evaluate a classifier’s performance, the most common criterion used in the literature is classifier accuracy defined as:

$$Accuracy=\frac{TP+TN}{TP+TN+FP+FN}$$

where $TP$ represents true positive, $TN$ represents true negative, $FP$ represents false positive, and $FN$ represents false negative rates. Accuracy estimates can be misleading if the number of trials of different classes are imbalanced. For instance, if an individual has great memory with few misses and a classifier labels all of the trials as remembered, although its accuracy will be high, classification is not informative since misses have not been identified (i.e., low specificity). Consequently, in order to take the imbalance between the number of trials of different classes into account, a modified version of accuracy—known as balanced accuracy—is often used (Brodersen, Ong, Stephan, & Buhmann, 2010; Kelleher, Namee, & D’Arcy, 2015):

$$Balanced accuracy=\frac{\frac{TP}{TP+FN}+\frac{TN}{TN+FP}}{2}$$

It is worth mentioning that balanced accuracy can be used similarly for multiclass classification problems.

Furthermore, to investigate whether a classifier has performed above the chance level, we used permutation tests (Nichols & Holmes, 2002) by repeating the classification analysis to reach an empirical null distribution for the classifier performance. Specifically, we carried out the same five-fold cross validation classification procedure but used labels that were randomly shuffled at each repetition. This process was conducted 500 times per analysis with random label assignment on each repetition. This established an empirical null distribution of classification performance according to balanced accuracy. Subsequently, we set the balanced accuracy, which was higher than 95% of the balanced accuracy values in the null distribution, as the threshold for determining the significance of a classifier’s performance.

*Impact of the number of features*

To choose how many features should be passed to the wrapper, we varied the size of the feature space and performed several classification analyses based on the number of features the wrapper searched through to select the best features for the classification of item hits versus misses. To be consistent, the wrapper always selected the best 10 features among the features through which it was searching. Moreover, to choose how many features the wrapper should select, we selected between 1 and 30 from the 100 filtered features. We performed these two analyses—the classification performance depending on how many features the wrapper searches through and how many features the wrapper selects— for 10 participants using all four classifiers we have used in this study. We chose 10 participants as these analyses were computationally expensive and doing it for all 60 participants for all analyses would be impractical. The results for naïve Bayes are shown in **Supplementary Figure 1**. The average patterns for the other classifiers were very similar (not shown).

Based on these two plots, in this study, it can be interpreted that filtering the top 100 features (40% of the trial numbers) for the wrapper to search through to select 10 effective features (4% of the trial numbers) among them seems to be an efficient choice for this dataset; this is because by filtering more than 100 features or selecting more than 10 features, time will be sacrificed considerably for only a slight increase in performance.

It is worth mentioning that while these plots are generalized based on the data size, a similar pattern would hold if we changed the horizontal axis to number of features/number of total extracted features instead.

**Supplementary Figure 1**. The relationship between **(a)** the classification’s performance and the number of features the wrapper searches through **(b)** the classification’s average running time and the number of features the wrapper searches through **(c)** the classification’s performance and the number of features the wrapper selects **(d)** the classification’s average running time and the number of features the wrapper selects. For the results to be generalizable across different problems, the horizontal axes represent the percent ratio of the number of features to data size—number of trials— instead of the actual number of features. Furthermore, the running time represents the average running time in second for classifying a trial.

*Different methods for handling class imbalance*

In this study, while we handled the imbalance issue using the synthetic minority oversampling method or “SMOTE”, we were also interested in examining how much controlling the imbalance could influence the performance, and whether an alternative strategy would affect performance. The alternative approach that we used is known as bootstrap aggregation or “bagging” in which we under-sampled from the class with more trials for 20 times so that the classifier was trained with equal number of trials for each class, and a vote was taken to generate the final test labels (Breiman, 1996; Kuhn & Johnson, 2013; Li, 2007). This approach was computationally costly and hence we applied it to 10 participants for classifying item recognition. Across different classifiers, we compared the performances of these two imbalance handling strategies with the control approach which is to not handle the imbalance at all. It is important to note that since the previously stated results indicated that selecting the effective features by the combination of filter and combination methods lead to higher performances, we only used that approach for this problem. The summary of the results can be found in **Supplementary** **Figure 2**.

**Supplementary Figure 2**. Comparisons of balanced accuracy and running time for different imbalance handling methods and classifiers for item recognition (hits vs. misses). **A)** balanced accuracy; and **B)** running time (in seconds per trial). The violin plots indicate the distribution of data scores. The box plots are shown inside the violin plots.

In order to statistically compare imbalance handling methods and classifiers, we ran an imbalance handling (SMOTE, bagging, no handling) $\times$ classifier (LASSO, logistic regression, naïve Bayes, and SVM) ANOVA for both balanced accuracy and running time. For balanced accuracy, significant main effect of imbalance handling [$F\left( 2,108 \right)=66.69, p<0.001, \eta_{p}^{2}= 0.553$] and the interaction $[F\left( 6,108 \right)=8.85, p<0.001, \eta_{p}^{2}= 0.330$] were observed. The main effect of classifier $[F\left( 3,108 \right)=2.25, p=0.087, \eta_{p}^{2}= 0.059]$ was not significant. As can be seen in **Supplementary** **Figure 2a,** follow-up t-tests showed that the SMOTE method outperformed the bagging method for all classifiers [all *ts* > 2.71, *ps* < 0.012] and outperformed the control method (i.e., not handling the imbalance) for all classifiers [all *ts* > 2.82, *ps* < 0.010] except naïve Bayes [*t*(9) < 0.001, *p* = 0.500]. Moreover, the control method outperformed the bagging method for naïve Bayes and logistic Regression [all *ts* > 5.55, *ps* < 0.001] while the bagging method outperformed the control method for SVM [*t*(9) = 1.87, *p* = 0.047]. No significant difference was found between these two methods for LASSO [*t*(9) = 0.96, *p* = 0.180].

For running time, main effects of imbalance handling $[F\left( 2,108 \right)=140.19, p<0.001, \eta_{p}^{2}= 0.722]$ and classifier $[F\left( 3,108 \right)=40.08, p<0.001, \eta_{p}^{2}= 0.527]$ and the interaction $[F\left( 6,108 \right)=21.14, p<0.001, \eta_{p}^{2}= 0.540]$ were all significant. Follow-up t-tests confirmed that the SMOTE and the control methods were significantly faster than bagging method for all classifiers [all *ts* > 2.92, *ps* < 0.009]. The control method was faster than SMOTE only for SVM [*t*(9) = 2.65, *p* = 0.013] but not for other classifiers [all *ts* < 1.67, *ps* > 0.065]. Moreover, as can be seen in **Supplementary** **Figure 2b**, naïve Bayes was faster than the others for the SMOTE method [all *ts* > 2.35, *ps*  < 0.022]. For bagging method, naïve Bayes was faster than the others [all *ts* > 6.14, *ps*  < 0.001] except SVM [*t*(9) = 1.26, *p* = 0.120]. Lastly, for the control method, SVM was faster than the others [all *ts* > 5.18, *ps*  < 0.001] except naïve Bayes [*t*(9) = 1.09, *p* = 0.152].

The significant interaction term in this problem indicates how each classifier can handle the imbalance issue by itself and without balancing the number of trials for each class. Specifically, naïve Bayes handles the imbalance very well as not handling the imbalance did not lead to a lower performance compared to handling imbalance using the SMOTE method. One of the reasons for this is that naïve Bayes does not solve an optimization problem to reduce the cost/error function. Specifically, when a classifier is adjusting its parameter to reduce the cost, it might label all of the trials as the class with majority of the trials to minimize the associated error function. However, since naïve Bayes does not do this, it is less sensitive to the imbalance (Ali, Shamsuddin, & Ralescu, 2015; Astrand et al., 2014; Daskalaki, Kopanas, & Avouris, 2006). On the other hand, SVM is very sensitive to imbalance, as can be seen in **Supplementary** **Figure 2a**.

Moreover, the fact that bagging has not performed well could be related to the number of trials that there were in our problem. Specifically, suppose an adult has good memory and remembers 220 items while forgets 20 of them. At each classification analysis, the bagging method ignores 200 remembered items (i.e., 83.3% of the information) and ends up with 40 trials which might not be sufficient for properly training the classification model. This issue can affect the performance even though the under-sampling will be repeated multiple times so that the classifiers are provided with different sets of trials from the overrepresented class and the aggregation of these classifiers could lead to a higher overall performance. Consequently, it is not surprising to see some classifiers (i.e., naïve Bayes and logistic Regression) perform better when they are provided with all the trials even though the imbalance issue is not handled (i.e., the control method).

*Different voting method approached for multiclass classification*

In this study, while we used the one against others approach for the voting method in multiclass classification, we were also interested in examining how the performance and running time would change if we used the one against one approach instead. We tried both methods for 10 participants for the 4-class context memory and the 3-class context perception problems. The summary of the results can be found in **Supplementary** **Figure 3**.

**Supplementary Figure 3**. Comparisons of balanced accuracy and running time for different voting methods and classifiers **A)** balanced accuracy; and **B)** running time (in seconds per trial) for context memory and **C)** balanced accuracy; and **D)** running time (in seconds per trial) for context perception. The violin plots indicate the distribution of data scores. The box plots are shown inside the violin plots.

For context memory classification, to statistically compare voting methods and classifiers, we ran a Voting method (one against others, one against one) $\times$ Classifier (LASSO, logistic regression, naïve Bayes, and SVM) ANOVA for both balanced accuracy and running time. For balanced accuracy, the main effect of Voting method [$F\left( 1,72 \right)=0.02, p=0.892, \eta_{p}^{2}< 0.001$], the main effect of Classifier $[F\left( 3,72 \right)=2.52, p=0.065, \eta_{p}^{2}= 0.095]$, and the interaction $[F\left( 3,72 \right)=0.08, p=0.973, \eta_{p}^{2}= 0.003$] were all non-significant.

For running time, main effects of Voting method $[F\left( 1,72 \right)=13.39, p=0.001, \eta_{p}^{2}= 0.157]$ and Classifier $[F\left( 3,72 \right)=55.08, p<0.001, \eta_{p}^{2}= 0.696]$ were significant while the interaction $[F\left( 3,72 \right)=1.81, p=0.154, \eta_{p}^{2}= 0.071]$ was not significant. as can be seen in **Supplementary** **Figure 3b**, the one against others approach was significantly faster than the one against one approach. This is not surprising as one against others performs $\binom{4}{1}$= 4 set of classifications while one against others performs $\binom{4}{2}=6$ set of classifications. Moreover, follow-up t-tests confirmed that naïve Bayes was faster than the others [all *ts* > 24.29, *ps*  < 0.002].

We did a similar set of analyses for context perception. For balanced accuracy, while the main effect of Voting method [$F\left( 1,72 \right)=0.01, p=0.916, \eta_{p}^{2}< 0.001$] and the interaction $[F\left( 3,72 \right)=0.06, p=0.982, \eta_{p}^{2}= 0.002$] were non-significant, the main effect of Classifier $[F\left( 3,72 \right)=3.02, p=0.035, \eta_{p}^{2}= 0.112]$ was significant. follow-up t-tests confirmed that naïve Bayes outperformed SVM [*t*(9) = 2.87, *p* = 0.009] but not others [all *ts* < 1.24, *ps*  > 0.123].

For running time, while the main effect of Voting method [$F\left( 1,72 \right)=3.78, p=0.056, \eta_{p}^{2}= 0.050$] and the interaction $[F\left( 3,72 \right)=0.06, p=0.978, \eta_{p}^{2}= 0.002$] were non-significant, the main effect of Classifier $[F\left( 3,72 \right)=58.49, p<0.001, \eta_{p}^{2}= 0.708]$ was significant. as can be seen in **Supplementary** **Figure 3d**, follow-up t-tests indicated that naïve Bayes was faster than the others [all *ts* > 9.62, *ps*  < 0.001].

*The importance of temporal resolution of extracted features*

In this study, we used a 200 ms temporal resolution for extracting features in this study i.e., the 400 ms time windows that we used for extracting features were sliding for 200 ms ([0 400], [200 600], [400 800], …, [1600 2000] ms). Given that one of the advantages of EEG is its temporal resolution, it could be a potential missed opportunity to not extract features from time windows with higher temporal resolution such as 20 ms (i.e., ([0 400], [20 420], [40 440], …, [1600 2000] ms). As a result, for the item memory problem, we conducted multiple classification analyses with different temporal resolutions for 10 participants to inspect how much the temporal resolution impacts the performance and running time. The obtained results are shown in **Supplementary** **Figure 4**. Although this figure is for naïve Bayes, the other classifiers showed a similar pattern.

**Supplementary Figure 4**. The relationship between **(a)** classifier performance and the temporal resolution for extracted the features **(b)** average running time and temporal resolution of extracted features.

As can be seen in this figure, by increasing the temporal resolution, while the performance will slightly increase, the running time significantly increases. As a result, choosing 200 ms as the temporal resolution was a reasonable choice.

*Comparison of feature selection methods in multiclass classification*

While we compared the effectiveness of the filter method and the combination of filter and wrapper methods for feature selection for the binary problems, we did not compare these methods for the multiclass problems in the main paper. Here, we compared these methods for the multiclass problems and the summary of the results can be found in **Supplementary** **Figure 5**. We did this analysis for all eligible participants for the 4-class context memory classification when the binary decision tree method was used for generalization. We repeated the same process for 10 subjects for the voting method and for the 3-class context perception problem and the results followed a similar pattern which are not shown here.

**Supplementary Figure 5**. Comparisons of **A)** balanced accuracy and **B)** running time (in seconds per trial) for different feature selection methods and classifiers for the 4-class context memory problem when the binary decision tree method was used for generalization. The violin plots indicate the distribution of data scores. The box plots are shown inside the violin plots.

To statistically compare feature selection methods and classifiers, we ran a Feature selection (filter, filter + wrapper) $\times$ Classifier (LASSO, logistic regression, naïve Bayes, and SVM) ANOVA for both balanced accuracy and running time. For balanced accuracy, significant main effect of Feature selection [$F\left( 1,224 \right)=1078.79, p<0.001, \eta_{p}^{2}= 0.828$] was found. The main effect of Classifier $[F\left( 3,224 \right)=2.63, p=0.051, \eta_{p}^{2}= 0.034]$ and the interaction $[F\left( 3,224 \right)=2.27, p=0.081, \eta_{p}^{2}= 0.029$] were not significant. As can be seen in **Supplementary** **Figure 5a**, the combination of filter and wrapper methods outperform the filter method.

For running time, main effects of Feature selection $[F\left( 1,224 \right)=174.13, p<0.001, \eta_{p}^{2}= 0.437]$ and Classifier $[F\left( 3,224 \right)=31.71, p<0.001, \eta_{p}^{2}= 0.298]$ and the interaction $[F\left( 3,224 \right)=28.17, p<0.001, \eta_{p}^{2}= 0.273]$ were all significant. Follow-up t-tests confirmed that the filter selection method was faster than the combination of filter and wrapper methods [all *t*s > 3.931, *p*s < 0.001]. As can be seen in **Supplementary** **Figure 5b**, naïve Bayes was faster than the others [all *t*s > 1.89, *p*s < 0.035] except SVM [*t*(28) = 1.39, *p* = 0.087] for the combination of filter and wrapper methods. For the filter method, naïve Bayes was faster than the others [all *t*s > 5.11, *p*s < 0.001].

*A summary of how frequently, different feature types, time intervals, frequency bands, and electrode regions were selected in the feature selection process*

**Table 1**. The distribution of the feature types for the selected features among all participants across each cognitive problem. It’s informative to note that for the first three problems, each of CSP features, means, variances, entropies, and phases included 17.0%, phases synchronies and correlations each included 3.2%, while AR model coefficients included 8.6% of the extracted features. For the EEG working memory problem, each of CSP features, means, variances, entropies, and phases included 18.4%, phases synchronies and correlations each included 1.7%, while AR model coefficients included 4.6% of the extracted features. For the motor imagery problem, each of CSP features, means, variances, entropies, and phases included 18.3%, phases synchronies and correlations each included 1.8%, while AR model coefficients included 4.9% of the extracted features. Lastly, for the MEG working memory problem, each of CSP features, means, variances, entropies, and phases included 19.5%, phases synchronies and correlations each included 0.6%, while AR model coefficients included 1.3% of the extracted features.

|  | CSP | Mean | Variance | Entropy | AR | Correlation | Phase | Phase synchrony |
| --- | --- | --- | --- | --- | --- | --- | --- | --- |
| Item memory | 70.6% | 7.9% | 0.6% | 4.3% | 1.1% | 1.5% | 11.9% | 2.1% |
| 3-class context decoding | 62.9% | 4.5% | 1.6% | 12.2% | 1.6% | 2.4% | 12.3% | 2.5% |
| 4-class context memory decoding | 61.8% | 3.7% | 1.5% | 13.0% | 1.5% | 2.6% | 12.9% | 3.0% |
| EEG working memory | 38.8% | 12.0% | 10.2% | 15.7% | 3.0% | 1.4% | 17.3% | 1.7% |
| Motor imagery | 70.1% | 0.0% | 3.2% | 8.1% | 0.0% | 0.0% | 17.9% | 0.7% |
| MEG working memory | 87.0% | 3.9% | 2.8% | 1.4% | 2.5% | 0.6% | 0.7% | 1.1% |

**Table 2**. The distribution of the time intervals (in milliseconds) for the selected features among all participants across each cognitive problem.

|  | [0-400] | [200-600] | [400-800] | [600-1000] | [800-1200] | [1000-1400] | [1200-1600] | [1400-1800] | [1600-2000] |
| --- | --- | --- | --- | --- | --- | --- | --- | --- | --- |
| Item memory | 8.4% | 10.3% | 12.5% | 12.7% | 12.5% | 10.7% | 13.3% | 10.4% | 9.2 |
| 3-class context perception | 12.1% | 12.6% | 11.3% | 11.1% | 12.1% | 10.5% | 9.7% | 10.3% | 10.3% |
| 4-class context memory decoding | 11.3% | 9.9% | 11.4% | 13.0% | 11.9% | 11.7% | 9.9% | 10.5% | 10.4% |
| EEG working memory | 11.7% | 10.2% | 11.1% | 12.6% | 11.9% | 11.4% | 10.2% | 10.7% | 10.2% |
| Motor imagery | 11.6% | 12.1% | 16.2% | 15.6% | 10.3% | 9.7% | 5.2% | 6.5% | 13.0% |
| MEG working memory | 13.9% | 14.7% | 16.7% | 16.1% | 9.1% | 8.7% | 7.5% | 7.3% | 6.0% |

**Table 3**. The distribution of the frequency bands for the selected features among all participants across each cognitive problem. Note that for the validation problems, the features were extracted from the EEG voltages.

|  | Theta | Alpha | Beta | Gamma |
| --- | --- | --- | --- | --- |
| Item memory | 34.2% | 25.2% | 27.5% | 13.1% |
| 3-class context perception | 37.2% | 31.7% | 23.9% | 7.2% |
| 4-class context memory decoding | 35.9% | 31.9% | 24.2% | 8.0% |

**Table 4**. The percentage of the selected electrodes from each electrode region for the selected features across all participants for each cognitive problem.

|  | Frontal left | Frontal right | Posterior left | Posterior right |
| --- | --- | --- | --- | --- |
| Item memory | 28.3% | 21.3% | 28.1% | 22.3% |
| 3-class context perception | 24.1% | 22.5% | 28.1% | 25.2% |
| 4-class context memory decoding | 24.8% | 20.4% | 28.0% | 26.8% |
| EEG working memory | 21.2% | 28.8% | 26.4% | 23.6% |
| Motor imagery | 17.1% | 41.5% | 13.8% | 27.6% |
| MEG working memory | 21.7% | 27.2% | 14.9% | 36.2% |

References

Ali, A., Shamsuddin, S. M., & Ralescu, A. (2015). *Classification with class imbalance problem : a review*. (August 2016).

Astrand, E., Enel, P., Ibos, G., Dominey, P. F., Baraduc, P., & Ben Hamed, S. (2014). Comparison of classifiers for decoding sensory and cognitive information from prefrontal neuronal populations. *PloS One*, *9*(1), e86314. https://doi.org/10.1371/journal.pone.0086314

Breiman, L. (1996). Bagging predictors. *Machine Learning*, *24*(2), 123–140. https://doi.org/10.1007/BF00058655

Brodersen, K. H., Ong, C. S., Stephan, K. E., & Buhmann, J. M. (2010). The Balanced Accuracy and Its Posterior Distribution. *2010 20th International Conference on Pattern Recognition*, 3121–3124. https://doi.org/10.1109/ICPR.2010.764

Daskalaki, S., Kopanas, I., & Avouris, N. (2006). Evaluation of Classifiers for an Uneven Class Distribution Problem. *Applied Artificial Intelligence*, *20*, 381–417. https://doi.org/10.1080/08839510500313653

Kelleher, J. D., Namee, B. Mac, & D’Arcy, A. (2015). *Fundamentals of Machine Learning for Predictive Data Analytics: Algorithms, Worked Examples, and Case Studies*. The MIT Press.

Kuhn, M., & Johnson, K. (2013). Applied Predictive Modeling. In *Applied Predictive Modeling.* https://doi.org/10.1007/978-1-4614-6849-3

Li, C. (2007). *Classifying imbalanced data using a bagging ensemble variation (BEV)*. https://doi.org/10.1145/1233341.1233378

.
